# 1700029I15Rik orchestrates the biosynthesis of acrosomal membrane proteins required for sperm–egg interaction

**DOI:** 10.1073/pnas.2207263120

**Published:** 2023-02-14

**Authors:** Yonggang Lu, Kentaro Shimada, Shaogeng Tang, Jingjing Zhang, Yo Ogawa, Taichi Noda, Hiroki Shibuya, Masahito Ikawa

**Affiliations:** ^a^Immunology Frontier Research Center, Osaka University, Osaka 565-0871, Japan; ^b^Department of Experimental Genome Research, Research Institute for Microbial Diseases, Osaka University, Osaka 565-0871, Japan; ^c^Graduate School of Pharmaceutical Sciences, Osaka University, Osaka 565-0871, Japan; ^d^Sarafan ChEM-H, Stanford University, Stanford, CA 94305; ^e^Department of Biochemistry, Stanford University School of Medicine, Stanford, CA 94305; ^f^Department of Chemistry and Molecular Biology, University of Gothenburg, Gothenburg SE-41390, Sweden; ^g^Division of Reproductive Biology, Institute of Resource Development and Analysis, Kumamoto University, Kumamoto 860-0811, Japan; ^h^Priority Organization for Innovation and Excellence, Kumamoto University, Kumamoto 860-8555, Japan; ^i^Laboratory of Reproductive Systems Biology, Institute of Medical Science, The University of Tokyo, Tokyo 108-8639, Japan; ^j^Center for Infectious Disease Education and Research, Osaka University, Osaka 565-0871, Japan

**Keywords:** 1700029I15Rik, C11orf94/Frey, sperm–egg fusion, fertilization, male infertility

## Abstract

Sperm acrosomal membrane proteins, such as Izumo sperm–egg fusion 1 (IZUMO1) and sperm acrosome-associated 6 (SPACA6), play essential roles in mammalian gamete binding or fusion. How their biosynthesis is regulated during spermiogenesis has largely remained elusive. Here, we show that *1700029I15Rik* knockout male mice are severely subfertile and their spermatozoa do not fuse with eggs. 1700029I15Rik is a type-II transmembrane protein expressed in early round spermatids but not in mature spermatozoa. It interacts with proteins involved in *N*-linked glycosylation, disulfide isomerization, and endoplasmic reticulum (ER)–Golgi trafficking, suggesting a potential role in nascent protein processing. The ablation of *1700029I15Rik* destabilizes non-catalytic subunits of the oligosaccharyltransferase (OST) complex that are pivotal for *N*-glycosylation. The knockout testes exhibit normal expression of sperm plasma membrane proteins, but decreased abundance of multiple acrosomal membrane proteins involved in fertilization. The knockout sperm show upregulated chaperones related to ER-associated degradation (ERAD) and elevated protein ubiquitination; strikingly, SPACA6 becomes undetectable. Our results support for a specific, 1700029I15Rik-mediated pathway underpinning the biosynthesis of acrosomal membrane proteins during spermiogenesis.

## Significance

In mammals, multiple sperm acrosomal membrane proteins are indispensable for sperm–egg interaction, yet the mechanisms underlying their biosynthesis have remained unknown. This study reveals that mouse 1700029I15Rik stabilizes OST subunits important for *N*-glycosylation and specifically mediates the processing of acrosomal membrane proteins in the ER of early round spermatids. We propose that during spermiogenesis, the biosynthesis of proteins destined for different subcellular compartments is orchestrated in a spatiotemporal manner. Given that 1700029I15Rik is highly conserved in humans, our discoveries in mice may provide profound insights into the etiology of idiopathic male infertility and the development of a nonhormonal contraceptive approach involving molecular interventions in the biosynthesis of acrosomal membrane proteins.

Mammalian fertilization is a complicated series of events culminating in the union of a haploid spermatozoon and a haploid egg to produce a diploid zygote. This process is a fundamental and obligatory prerequisite for successful propagation of parental genomes to the next generation. During the journey through the female reproductive tract, spermatozoa undergo the acrosome reaction to expose the acrosomal contents, including hydrolytic enzymes and acrosomal membrane proteins, to gain competence in penetrating the egg zona pellucida (ZP) and fusing with the oolemma (1, 2).

Gamete fusion is a fascinating eukaryotic cell–cell fusion event involving the merger of two morphologically distinct cells from individual organisms of opposite genders. Notwithstanding the decades-long research effort, detailed cellular and molecular mechanisms underlying this unique fusion process remain shrouded in mystery (2). To date, seven sperm proteins, Izumo sperm–egg fusion 1 (IZUMO1) (3, 4), sperm acrosome-associated protein 6 (SPACA6) (5–7), fertilization-influencing membrane protein (FIMP) (8), sperm–oocyte fusion-required 1 (SOF1) (6), transmembrane protein 95 (TMEM95) (6, 9, 10), and DC-STAMP domain-containing 1 and 2 (DCST1 and DCST2) (11, 12), have been demonstrated essential for sperm–egg binding or fusion. Mouse sperm lacking any of these molecules fail to fuse with the oolemma, despite their normal morphology, motility, and the ability to elicit the acrosome reaction. Among these proteins, IZUMO1, SPACA6, TMEM95, DCST1, and DCST2 are transmembrane glycoproteins that initially localize to the acrosomal membrane and translocate to the sperm surface during the acrosome reaction (3, 6, 12, 13).

In the early spermatids, many acrosomal protein precursors are synthesized and processed in the endoplasmic reticulum (ER), trafficked to the Golgi apparatus, and secreted as proacrosomal vesicles that subsequently coalesce to form the acrosome (14). In this study, by harnessing genetics, proteomics, and cell biology approaches, we unraveled that 1700029I15Rik, a type-II transmembrane protein, facilitates the biosynthesis of acrosomal membrane proteins involved in sperm–egg interaction.

## Results

### 1700029I15Rik Is a Testis-Specific Type-II Transmembrane Protein Expressed during Early Spermiogenesis.

**Fig. 1.**
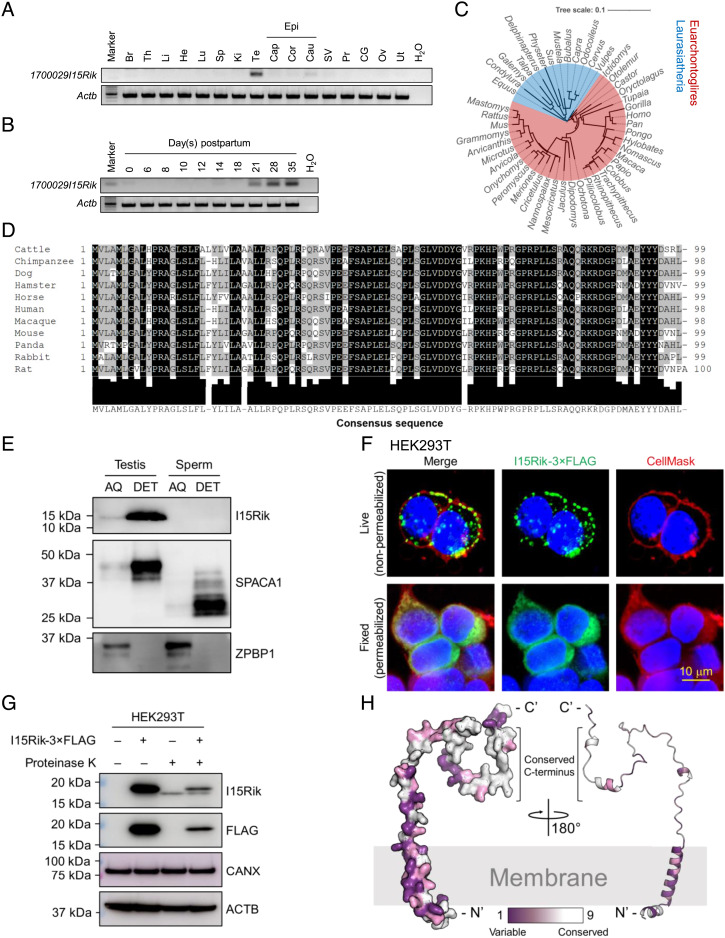
*1700029I15Rik* Is a Testis-Enriched Type-II Transmembrane Protein Conserved in Mammals and Expressed during Spermiogenesis. (*A*) Analysis of *1700029I15Rik* expression in various mouse tissues by RT-PCR. Br, brain; Th, thymus; Li, liver; He, heart; Lu, lung; Sp, spleen; Ki, kidney; Te, testis; Cap, caput epididymis; Cor, corpus epididymis; Cau, cauda epididymis; Epi, epididymis; SV, seminal vesicle; Pr, prostate; CG, coagulating gland; Ov, ovary; Ut, uterus. The expression of *β-actin* (*Actb*) was analyzed as a loading control. (*B*) Analysis of *1700029I15Rik* expression in mouse testes during postnatal development. (*C*) Phylogenetic tree depicting the evolutionary conservation of 1700029I15Rik in mammals. The tree was visualized using the interactive Tree of Life (iTOL) (16). Red and blue highlighted species belong to Euarchontoglires and Laurasiatheria, respectively. (*D*) Multiple sequence alignment of 1700029I15Rik orthologous proteins in 11 mammalian species. The *Lower* panel indicates the consensus sequence and the extent of amino acid conservation. (*E*) Western blot detection of 1700029I15Rik (I15Rik) in mouse testes and sperm fractionated by Triton X-114. SPACA1 and zona pellucida binding protein 2 (ZPBP1) were analyzed as positive controls for the proteins enriched in the AQ and DET phases, respectively. (*F*) In vitro topological analysis of 1700029I15Rik by live cell immunostaining. HEK293T cells were transiently transfected with a plasmid encoding C-terminal 3 × FLAG-tagged 1700029I15Rik. Live or fixed HEK293T cells were probed with an anti-FLAG antibody and an Alexa Fluor™ 488-conjugated secondary antibody. Cell membranes and nuclei were visualized by CellMask™ deep red plasma membrane stain and Hoechst 33342, respectively. (*G*) In vitro proteinase K protection assay depicting the topology of 1700029I15Rik. Live HEK293T cells transiently expressing 1700029I15Rik-3 × FLAG were treated with proteinase K and subjected to protein extraction. The levels of 1700029I15Rik-3 × FLAG before and after the enzyme treatment were analyzed by Western blotting. CANX and ACTB were analyzed in parallel as loading controls. (*H*) 1700029I15Rik protein structure predicted by AlphaFold (AF-Q8CF31-F1) (17). The degree of residue conservation was determined by ConSurf (18) and plotted to the three-dimensional structures, with purple representing variable and white representing conserved.

*1700029I15Rik* encompasses three coding exons and is localized to the forward strand of mouse chromosome 2 (*SI Appendix*, Fig. S1*A*). This gene resides on chromosome 11 in humans, thus named *C11orf94*. RT-PCR revealed that *1700029I15Rik* exhibits a testis-biased expression, which is initiated at postnatal day 21 (Fig. 1 *A* and *B*), corresponding to the first wave of mouse spermiogenesis. Consistently, a previously published single-cell RNA-sequencing (scRNA-seq) analysis (15) indicated that *1700029I15Rik* expresses predominantly in mid-round spermatids (*SI Appendix*, Fig. S1*B*). 1700029I15Rik is conserved among mammals (Fig. 1 *C* and *D* and *SI Appendix*, Fig. S1*C*). In certain vertebrates, such as black flying foxes and green sea turtles, the C terminus of peroxisomal biogenesis factor 16 (PEX16) is highly homologous to 1700029I15Rik (*SI Appendix*, Fig. S1 *D*–*F*). Given that *Pex16* is immediately upstream of *1700029I15Rik* in the genome (*SI Appendix*, Fig. S1*A*), we speculate that these species harbor a *Pex16*–*1700029I15Rik* chimeric fusion transcript.

1700029I15Rik contains a hydrophobic N-terminal region [amino acids (aa) 1 to 29], predicted as a transmembrane helix or a signal peptide by various algorithms (*SI Appendix*, Table S1 and Fig. S1*G*). For immunodetection of 1700029I15Rik, we produced a polyclonal antibody against its C terminus [aa 81 to 99 (*SI Appendix*, Fig. S1*G*)]. Triton X-114 subcellular fractionation unveiled that 1700029I15Rik is a membrane-bound protein exclusively detected in the detergent (DET) phase of testis lysates (Fig. 1*E*). To gain insights into the topology of 1700029I15Rik, we transiently transfected human embryonic kidney 293T (HEK293T) cells with a plasmid encoding C-terminal 3 × FLAG-tagged 1700029I15Rik, whose expression was validated by Western blotting (*SI Appendix*, Fig. S1*H*). By probing live HEK293T cells with an anti-FLAG antibody and a fluorophore-conjugated secondary antibody, we detected the fluorescence signal of 1700029I15Rik-3 × FLAG at the cell plasma membrane that was concomitantly labeled by CellMask™ plasma membrane stain (Fig. 1*F*). Likewise, in nonpermeabilized CV-1 in Origin Simian-7 (COS-7) monkey kidney cells, 1700029I15Rik is colocalized with the type-I transmembrane protein, IZUMO1, at the cell surface (*SI Appendix*, Fig. S1*I*). Since antibodies cannot diffuse through intact membranes, the C terminus of 1700029I15Rik is likely exposed extracellularly. To confirm this observation, we further treated live HEK293T cells overexpressing 1700029I15Rik-3 × FLAG with proteinase K, which nonspecifically cleaves cell surface proteins (19). After the enzyme treatment, the level of 1700029I15Rik was significantly reduced (Fig. 1*G*), indicating that the epitopes at the C terminus were proteolyzed. Taken together, these analyses demonstrate that 1700029I15Rik is a type-II transmembrane protein that is anchored to the lipid membrane via its N terminus (Fig. 1*H*).

### *1700029I15Rik* Knockout Male Mice Are Infertile due to Defective Sperm–Egg Interaction.

**Fig. 2.**
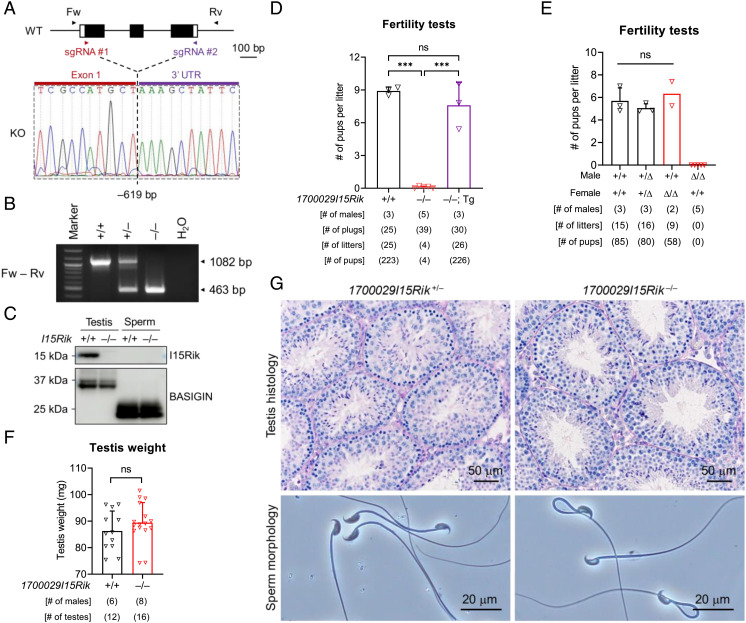
*1700029I15Rik* Knockout Males Are Infertile despite Normal Spermatogenesis and Sperm Morphology. (*A*) CRISPR/Cas9-mediated knockout of *1700029I15Rik* in the B6D2F1 hybrid mouse strain. Single guide RNAs (sgRNAs) #1 and #2 were designed to target the first coding exon (Exon 1) and the 3′ untranslated region (UTR), respectively. Forward (Fw) and reverse (Rv) primers flanking the deletion region were employed for genotyping. (*B*) Genomic PCR for detecting wild-type and *1700029I15Rik* knockout alleles. (*C*) Western blot detection of 1700029I15Rik in wild-type and knockout testes and sperm. BASIGIN was analyzed in parallel as a loading control. (*D*) Fertility tests of wild-type, *1700029I15Rik*^–/–^, and *1700029I15Rik*^–/–^; Tg male mice. ns, not significant. (*E*) Fertility tests of wild-type, *1700029I15Rik*^+/Δ^, and *1700029I15Rik*^Δ/Δ^ mice. The mutant mouse line was produced on an inbred genetic background of C57BL/6J. (*F*) Analysis of testis weight in wild-type and *1700029I15Rik*^–/–^ males. (*G*) Analyses of testis histology and sperm morphology in *1700029I15Rik*^+/–^ and *1700029I15Rik*^–/–^ males.

To examine the function of *1700029I15Rik* in vivo, we generated a knockout mouse line (*1700029I15Rik*^–/–^) on a hybrid background of B6D2F1 by CRISPR/Cas9 (Fig. 2*A* and *SI Appendix*, *SI Materials and Methods*). A 619-bp deletion was introduced to the coding region of *1700029I15Rik*, resulting in translation of an incorrect amino acid sequence (Fig. 2 *A* and *B* and *SI Appendix*, Fig. S2*A*). By Western blotting, we confirmed the absence of 1700029I15Rik in knockout testes (Fig. 2*C*). 1700029I15Rik is not detectable in wild-type sperm (Figs. 1*E* and 2*C*), implying its potential role restricted in spermatogenesis.

In parallel, we produced *1700029I15Rik* mutant mice (*1700029I15Rik*^Δ/Δ^) lacking 215 bp at the first coding exon on an inbred background of C57BL/6J (*SI Appendix*, Fig. S2 *B* and *C*). Both the knockout and mutant mice show normal development, appearance, and behavior. *1700029I15Rik*^–/–^ female mice exhibit normal fecundity, whereas *1700029I15Rik*^–/–^ males are severely subfertile despite successful coituses (*SI Appendix*, Fig. S2 *D*–*F*). A transgene (Tg) encoding C-terminal PA-tagged 1700029I15Rik restores the fecundity of knockout males (Fig. 2*D* and *SI Appendix*, Fig. S2 *G–I*), demonstrating that the male infertility is not caused by potential off-target mutations. *1700029I15Rik*^Δ/Δ^ males are sterile (Fig. 2*E*), suggesting that the phenotype severity might correlate with the genetic background. The testis weights, spermatogenesis, sperm morphology, and sperm counts are normal in *1700029I15Rik*^–/–^ males (Fig. 2 *F* and *G* and *SI Appendix*, Fig. S2*J*). Similarly, *1700029I15Rik*^Δ/Δ^ males show no abnormalities in the testis and epididymis histology, as well as the first meiotic division of spermatocytes (*SI Appendix*, Fig. S2 *K*–*M*). To avoid unnecessary duplication of efforts, the subsequent experiments were performed on *1700029I15Rik*^–/–^ mice.

**Fig. 3.**
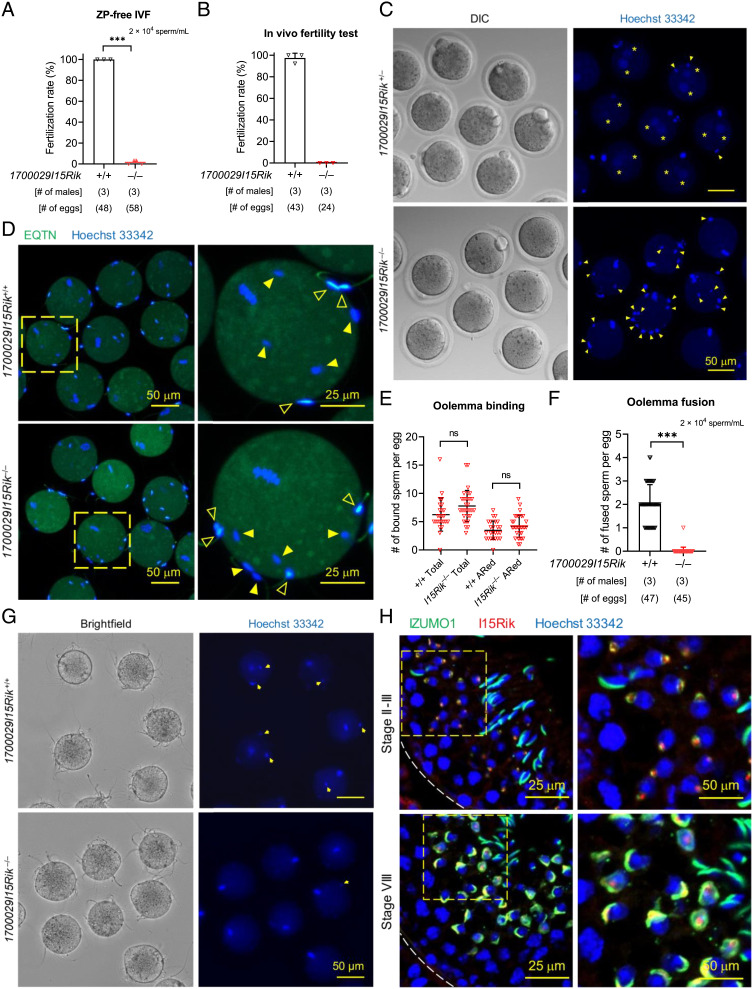
Infertility of *1700029I15Rik* Knockout Males Is Attributed to Impaired Sperm–Egg Interaction. (*A*) In vitro fertilization (IVF) analysis of sperm fertilizing ability using wild-type ZP-free eggs. (*B* and *C*) In vivo fertility test of wild-type and *1700029I15Rik*^–/–^ males. Eggs were harvested from superovulated B6D2F1 female mice that had copulated with wild-type or *1700029I15Rik* knockout males. Sperm in the perivitelline space (yellow arrowheads) and pronuclei in the fertilized eggs (yellow asterisks) were visualized by Hoechst 33342. (*D* and *E*) In vitro analysis of sperm–egg binding. Spermatozoa pre-incubated in the Toyoda, Yokoyama, Hoshi (TYH) medium were probed with an anti-EQTN antibody and an Alexa Fluor™ 488-conjugated secondary antibody to reveal the acrosomal status. The acrosome-intact and acrosome-reacted sperm are marked by solid and hollow arrowheads, respectively. Sperm heads were stained with Hoechst 33342. (*F* and *G*) In vitro analysis of sperm–egg fusion using Hoechst 33342-preloaded ZP-free eggs. Yellow arrows indicate the fused sperm heads carrying the Hoechst dye transferred from the eggs. (*H*) Coimmunostaining of IZUMO1 (green) and 1700029I15Rik (red) in wild-type testis cryosections. Cell nuclei were visualized by Hoechst 33342.

In vitro fertilization analyses revealed that *1700029I15Rik* knockout sperm showed significantly reduced ability to fertilize cumulus-intact, cumulus-free, and ZP-free eggs (*SI Appendix*, Fig. S3 *A* and *B* and Fig. 3*A*). The knockout sperm were unable to fertilize eggs in vivo and accumulated in the perivitelline space, indicating that they underwent the acrosome reaction and penetrated the ZP but failed to fuse with the eggs (Fig. 3 *B* and *C* and *SI Appendix*, Fig. S3*C*). Through sperm–egg binding and fusion assays, we confirmed that *1700029I15Rik* knockout sperm bind but do not fuse with the oolemma (Fig. 3 *D*–*G* and *SI Appendix*, *SI Materials and Methods*). Despite the impaired fertilizing ability, the knockout sperm exhibited normal motility and ability to bind the ZP (*SI Appendix*, Fig. S3 *D–H*).

Immunohistochemistry revealed that 1700029I15Rik initially appears at the center of the proacrosomal vacuole in steps 2 and 3 spermatids, distinct to IZUMO1 that localizes to the proacrosomal membrane (Fig. 3*H*). In step 8 spermatids, 1700029I15Rik is partially colocalized with IZUMO1 in the proacrosome. Nevertheless, 1700029I15Rik, but not IZUMO1, was detected in the acrosomal granule (Fig. 3*H*). From step 9 spermatids, 1700029I15Rik becomes undetectable in the proacrosome (*SI Appendix*, Fig. S4*A*). The manchette staining is nonspecific, because the same pattern of staining was observed in the knockout spermatids (*SI Appendix*, Fig. S4*B*). Together, these findings suggest that 1700029I15Rik, which is predominantly expressed in early spermatids, indirectly promotes sperm–egg interaction. In agreement with this assumption, HEK293T cells transiently expressing 1700029I15Rik could not bind or fuse with ZP-free mouse eggs (*SI Appendix*, Fig. S4 *C* and *D*).

### 1700029I15Rik Interacts with ER-Resident Proteins Implicated in Nascent Protein Processing and Stabilizes the OST Complex Subunits In Vivo.

**Fig. 4.**
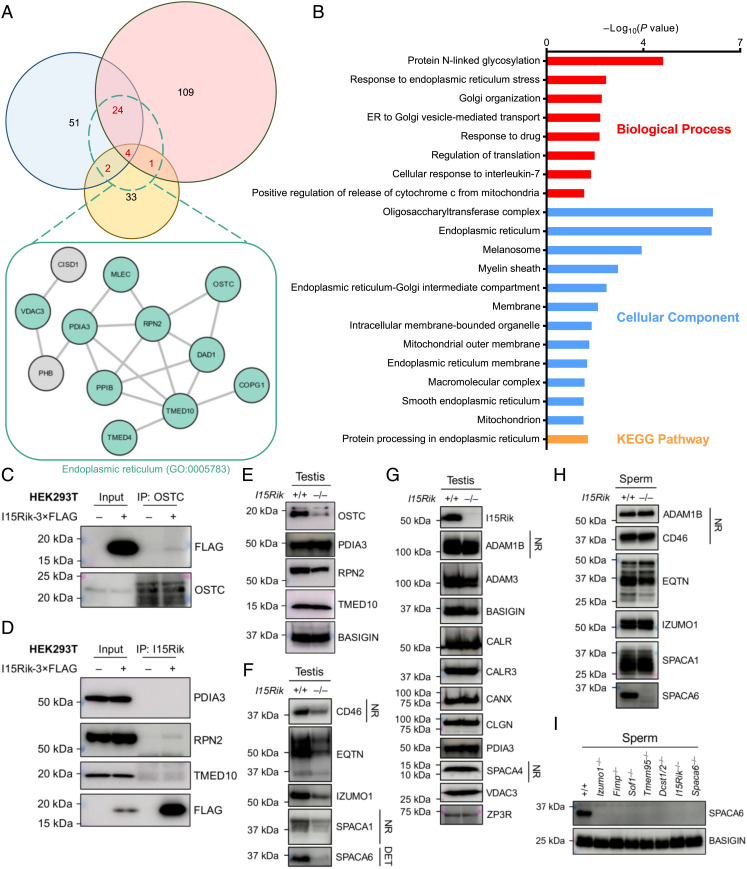
1700029I15Rik Interacts with Proteins Involved in *N*-glycosylation, Disulfide Bond Formation, and Vesicular Trafficking and Facilitates the Expression of Multiple Acrosomal Membrane Proteins. (*A*) Venn diagram depicting shared and unique protein hits identified in three biological replicates of co-IP/MS experiments. Only the proteins specifically detected in the wild-type samples are included. The proteins detected in at least two replicates were subjected to STRING analysis (20, 21). For each replicate, 1700029I15Rik and its interacting proteins were coimmunoprecipitated from wild-type and knockout testis lysates using antibody-crosslinked agarose resin. (*B*) GO and KEGG analyses of 1700029I15Rik-interacting proteins. Functional annotations were conducted using the Database for Annotation, Visualization and Integrated Discovery (DAVID) (22). Only the 31 proteins concurrently detected in at least two replicates of the co-IP/MS experiments were analyzed. (*C* and *D*) In vitro validation of protein–protein interactions. 1700029I15Rik and its associated proteins were coimmunoprecipitated from the lysates of HEK293T cells transiently expressing 1700029I15Rik-3 × FLAG using antibody-conjugated magnetic beads. The co-IP products were subjected to SDS-PAGE and Western blot analyses to validate protein–protein interactions. (*E*) Western blot analyses of OSTC, PDIA3, RPN2, and TMED10 in wild-type and knockout testes. BASIGIN was analyzed in parallel as a loading control. (*F* and *G*) Western blot analyses of various proteins in wild-type and knockout testes. Unless specified otherwise, all protein samples were processed under reducing and denaturing conditions. NR, non-reducing and non-denaturing. The DET fractions of wild-type and knockout testis proteins, extracted by Triton X-114, were used for immunodetection of SPACA6. (*H*) Western blot analyses of various membrane proteins in wild-type and knockout sperm. (*I*) Western blot detection of SPACA6 in the sperm of wild-type, *Izumo1*^–/–,^ *Fimp*^–/–,^ *Sof1*^–/–,^ *Tmem95*^–/–,^ *Dcst1/2*^–/–,^*1700029I15Rik*^–/–^, and *Spaca6*^–/–^ male mice. BASIGIN was analyzed as a loading control.

To identify the interactome of 1700029I15Rik in mouse testes, we next performed coimmunoprecipitation tandem mass spectrometry (co-IP/MS) analyses using antibody-crosslinked agarose resin (*SI Appendix*, Fig. S5*A* and *SI Materials and Methods*). As a result, 31 proteins, including 1700029I15Rik, were specifically detected in the wild-type samples from at least two of three biological replicates (Fig. 4 *A* and Dataset S1). Gene ontology (GO) and Kyoto Encyclopedia of Genes and Genomes (KEGG) analyses revealed that the 31 hits include proteins pivotal for *N*-glycosylation [e.g., defender against cell death 1 (DAD1), oligosaccharyltransferase complex noncatalytic subunit (OSTC), and ribophorin II (RPN2)] and ER–Golgi vesicular trafficking [e.g., transmembrane p24 trafficking protein 4 and 10 (TMED4 and TMED10); Fig. 4 *A* and *B*].

For determining 1700029I15Rik-interacting proteins with high confidence, another co-IP/MS analysis using non-crosslinked magnetic beads was conducted, and the relevance of protein–protein interactions was scored based on the specificity of detected spectra (*SI Appendix*, Fig. S5 *B*–*D*). Apart from the above-mentioned proteins, signal peptide peptidase like 2C (SPPL2C), protein disulfide isomerase family A member 3 (PDIA3), and voltage-dependent anion channel 3 (VDAC3) are also tightly associated with 1700029I15Rik (*SI Appendix*, Fig. S5*D*). SPPL2C is an intramembrane protease, homologous to signal peptide peptidases, and specific for type-II-oriented transmembrane proteins (23), but it is dispensable for male fertility (24). Interestingly, the level of 1700029I15Rik is dramatically decreased in *Sppl2c* knockout testes (23), suggesting that SPPL2C directly or indirectly governs the stability of 1700029I15Rik. PDIA3 is a protein disulfide isomerase that modulates protein folding, formation and remodeling of disulfide bonds, and quality control of glycoproteins (25). It might be implicated in sperm ZP binding and oolemma fusion, as revealed by previous inhibitory studies (26, 27).

We failed to verify whether OSTC, PDIA3, RPN2, or TMED10 interacts with 1700029I15Rik in testes by co-IP coupled with Western blotting (*SI Appendix*, Fig. S6*A*). This is likely owing to the low abundance of immunoprecipitated 1700029I15Rik and its interacting proteins, considering that no unique protein bands were detected in the silver-stained wild-type eluates (*SI Appendix*, Fig. S5 *A* and *B*). Alternatively, we overexpressed 1700029I15Rik-3 × FLAG in HEK293T cells and discovered that 1700029I15Rik interacts with the endogenous OSTC and RPN2, but not PDIA3 or TMED10 (Fig. 4 *C* and *D*). Strikingly, OSTC and RPN2, but not PDIA3 or TMED10, are significantly downregulated in the knockout testes (Fig. 4*E*). Nonetheless, immunohistochemistry revealed no obvious anomalies in the localization of the remaining OSTC in the knockout testes (*SI Appendix*, Fig. S6*B*). These findings indicate that 1700029I15Rik interacts with and stabilizes the OST complex subunits in the male germline.

### Depletion of *1700029I15Rik* Results in Reduced Expression of Multiple Acrosomal Membrane Glycoproteins.

Our co-IP/MS experiments suggest potential involvement of 1700029I15Rik in protein processing and trafficking. Given the impaired fertilizing ability of *1700029I15Rik* knockout sperm, we next examined the expression of fertilization-related proteins in testes and sperm by Western blotting. Interestingly, the amounts of acrosomal membrane *N*-linked glycosylated proteins, including membrane cofactor protein (CD46), equatorin (EQTN), IZUMO1, sperm acrosome-associated 1 (SPACA1), and SPACA6 are drastically reduced in the knockout testes (Fig. 4*F* and *SI Appendix*, Fig. S6 *C–E*). In contrast, the levels of sperm head plasma membrane proteins [e.g., a disintegrin and metallopeptidase domain 1b and 3 (ADAM1B and ADAM3)], ER chaperones [e.g., calreticulin (CALR), calnexin (CANX), and PDIA3], sperm acrosome-associated 4 (SPACA4), a glycosylphosphatidylinositol (GPI)-anchored protein localized to the acrosomal membrane, and zona pellucida 3 receptor (ZP3R), a soluble protein expressed in the acrosomal matrix, are comparable in wild-type and knockout testes (Fig. 4*G*). Noticeably, the downregulated acrosomal membrane proteins are all highly expressed in mid-round spermatids, whilst the plasma membrane proteins show peak expression in spermatocytes (*SI Appendix*, Fig. S6*F*). These findings unveil that 1700029I15Rik may specifically regulate the biosynthesis of acrosomal membrane glycoproteins expressed during spermiogenesis. Co-IP and Western blot analyses indicated that 1700029I15Rik exhibits no detectable interactions with IZUMO1 or EQTN in testes (*SI Appendix*, Fig. S6*G*). Transgenic expression of 1700029I15Rik-PA, although at a trace amount, restores the amount of IZUMO1 to the wild-type level (*SI Appendix*, Fig. S7*A*). Interestingly, CD46, EQTN, IZUMO1, and SPACA1, which are downregulated in the knockout testes, exhibited normal expression levels in the knockout sperm (Fig. 4*H*). Moreover, these acrosomal membrane proteins show normal subcellular localization in the knockout testes and sperm (*SI Appendix*, Fig. S7 *B* and *C*). SPACA6, however, is absent in *1700029I15Rik* knockout spermatozoa (Fig. 4*H* and *SI Appendix*, Fig. S7*D*), providing a plausible explanation for the defective oolemma fusion ability.

A previous study has indicated that SPACA6 is absent in *Izumo1*, *Dcst1*, and *Dcst2* knockout sperm (11). Here, we found that SPACA6 is also missing in *Tmem95*, *Fimp*, and *Sof1* knockout sperm (Fig. 4*I*). In the sperm of *Izumo1*^–/–^; Tg (*Izumo1-mCherry*) male mice, the amount of transgenically expressed IZUMO1 is lower than that of the wild-type protein. Intriguingly, the transgene does not fully restore SPACA6 to its endogenous level (*SI Appendix*, Fig. S7*E*). A similar phenomenon was observed in the sperm of *Tmem95*^–/–^; Tg (*Tmem95-1D4*) males (*SI Appendix*, Fig. S8*A*). RT-PCR revealed that the mRNA expression of *Spaca6* is normal in *Tmem95*^–/–^ and *Tmem95*^–/–^; Tg testes (*SI Appendix*, Fig. S8*B*). Based on these results, we propose that the presence and abundance of SPACA6 is dependent upon that of the other gamete fusion-related proteins. Further, the expression of 1700029I15Rik is normal in *Izumo1* and *Spaca6* knockout testes (*SI Appendix*, Fig. S8 *C* and *D*). These findings indicate that the loss of SPACA6 in *170029I15Rik* knockout males may be directly caused by a lack of the 1700029I15Rik-mediated protein biosynthesis pathway, or indirectly induced by aberrant expression of other gamete fusion-related acrosomal membrane proteins.

### *1700029I15Rik* Knockout Sperm Show Elevated Levels of ER Chaperones and Ubiquitination.

**Fig. 5.**
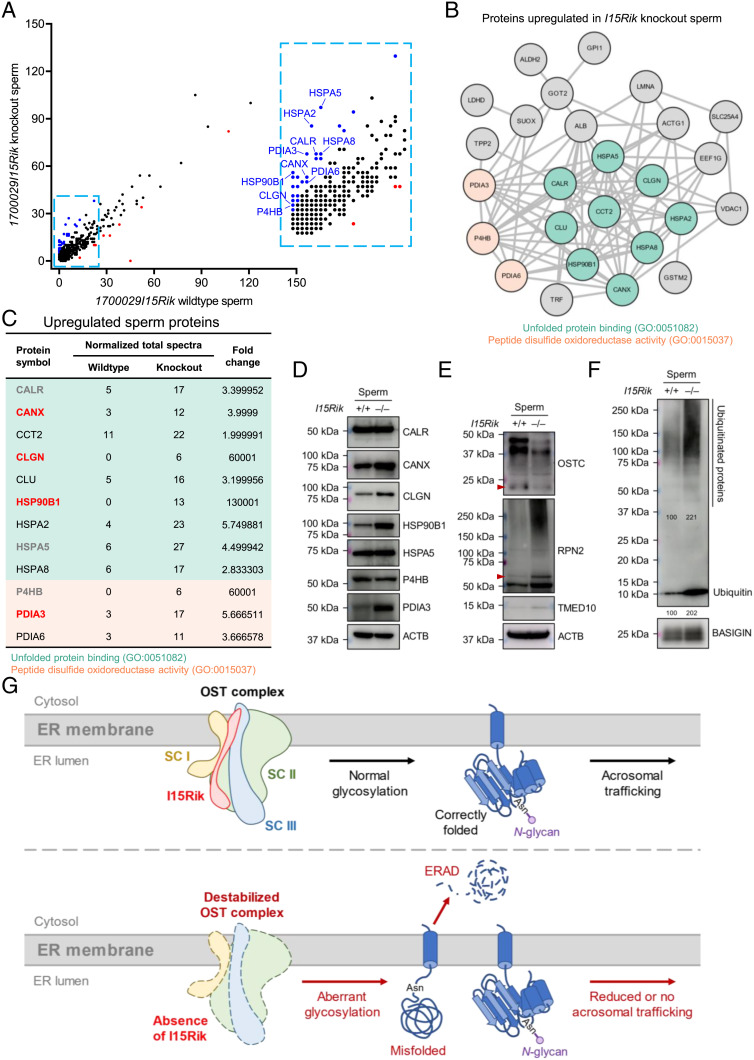
Ablation of *1700029I15Rik* Results in Upregulated ER Chaperones and Ubiquitinated Proteins in the Sperm. (*A*) MS analysis of wild-type and *1700029I15Rik* knockout sperm proteome. Blue and red dots indicate proteins upregulated and downregulated in the knockout spermatozoa, respectively. (*B*) STRING (20, 21) and GO (22) analyses of proteins upregulated in *1700029I15Rik* knockout spermatozoa. The proteins related to unfolded protein binding (GO:0051082) or exhibited peptide disulfide oxidoreductase activity (GO:0015037) are highlighted in teal and light orange, respectively. (*C*) Upregulated ER chaperones in *1700029I15Rik* knockout spermatozoa. The upregulation of CANX, CLGN, HSP90B1, and PDIA3 (highlighted in bold font and red color), but not CALR, HSPA5, and P4HB (in bold font and gray color), have been verified by Western blot analyses shown in Fig. 5*D*. (*D*) Western blot analyses of multiple ER chaperones in wild-type and knockout spermatozoa. ACTB was analyzed as a loading control. (*E*) Western blot analyses of OSTC, RPN2, TMED10 in wild-type and knockout spermatozoa. ACTB was analyzed as a loading control. (*F*) Western blot detection of ubiquitin and ubiquitinated proteins in wild-type and knockout spermatozoa. BASIGIN was analyzed in parallel as a loading control. The band intensities relative to BASIGIN were measured by ImageJ. The numbers represent the relative intensities of ubiquitinated proteins (*Upper*) or ubiquitin (*Lower*). (*G*) A diagram depicting 1700029I15Rik-mediated processing of acrosomal membrane proteins. OST complex is composed of subcomplex (SC) I, II, and III. Briefly, 1700029I15Rik interacts with and stabilizes OSTC and RPN2, which localize to SC II and SC III, respectively (28). In the absence of 1700029I15Rik, the destabilized OST complex causes aberrant glycosylation of acrosomal membrane proteins, resulting in increased protein misfolding. The misfolded proteins are eliminated by ubiquitin-dependent ERAD. Thus, the acrosomal trafficking of mature proteins is reduced or impaired.

We hypothesize that apart from SPACA6, there might be other acrosomal membrane glycoproteins downregulated in response to the absence of 1700029I15Rik. Considering that whole testis lysates are extremely heterogeneous that impedes the detection of low-abundance proteins by proteomics, we performed an MS analysis to compare the proteome of wild-type and knockout spermatozoa. As a result, we detected 35 proteins significantly upregulated and nine proteins significantly downregulated in the knockout sperm (Fig. 5*A*). GO and STRING analyses indicated that most of the upregulated proteins are ER chaperones that bind unfolded or misfolded proteins and coordinate the ubiquitin-dependent ER-associated degradation (ERAD) pathway [GO:0051082 and GO:0030433; e.g., CANX, CALR, and heat shock protein 90 beta family member 1 (HSP90B1)], or catalyze disulfide formation or remodeling [GO:0015037; e.g., prolyl 4-hydroxylase subunit beta (P4HB), PDIA3, and protein disulfide isomerase family A member 6 (PDIA6)] (Fig. 5 *B* and *C* and *SI Appendix*, Fig. S8*E*). The nine proteins downregulated in the knockout sperm, however, do not show distinct commonalities in their molecular functions; yet, acrosin (ACR), dipeptidase 3 (DPEP3), and inactive serine protease 39 (PRSS39), are associated with the acrosomal vesicle (GO:0001669; *SI Appendix*, Fig. S8*F*). Notably, the levels of EQTN, IZUMO1, and SPACA1 are slightly but non-significantly reduced in the knockout sperm, compared with those in wild-type sperm (*SI Appendix*, Fig. S8*G*). By Western blotting, we confirmed the upregulation of CANX, CLGN, HSP90B1, and PDIA3 in *1700029I15Rik* knockout spermatozoa; however, the levels of CALR, heat shock protein 5 (HSPA5), and P4HB are comparable between wild-type and knockout groups (Fig. 5*D*). Regarding the potential interactome of 1700029I15Rik, RPN2 and TMED10 are upregulated, whilst OSTC shows reduced abundance in the knockout sperm (Fig. 5*E*).

Concerning that low-abundance proteins might not be readily detected by MS in whole sperm lysates, we performed individual analyses on Triton X-114-extracted soluble and membrane fractions of wild-type and knockout sperm. Interestingly, multiple protein bands were specifically detected in the aqueous (AQ) phase, but not the DET phase of knockout sperm (*SI Appendix*, Fig. S9*A*). The MS analyses collectively uncovered 50 significantly upregulated and 34 significantly downregulated proteins in *1700029I15Rik* knockout sperm (Dataset S1). In agreement to the whole sperm proteomic analysis, multiple ERAD-associated chaperones are increased in the knockout sperm (*SI Appendix*, Fig. S9*B*). Apart from these chaperones, the OST complex subunits, ribophorin I (RPN1) and RPN2, and the ER–Golgi intermediate compartment (ERGIC) proteins, TMED4 and TMED10, are also upregulated in the knockout sperm (*SI Appendix*, Fig. S9*B*). GO and KEGG analyses revealed that the downregulated hits include proteins exhibited oxidoreductase activity (GO:0016491; 4 proteins), involved in the biosynthesis of amino acids (KEGG: mmu01230; 4 proteins), or associated with the acrosomal vesicle (GO:0001669; 4 proteins) or mitochondria (GO:0005739; 13 proteins). Notably, renalase (RNLS) and succinate-Coenzyme A ligase, ADP-forming, beta subunit (SUCLA2) are downregulated in both trials of sperm proteomic analyses (*SI Appendix*, Figs. S8*F* and S9*B*). However, whether these proteins play important roles in protein biosynthesis or fertilization remains unknown.

The increased ER chaperones involved in the unfolded protein response and ubiquitin-dependent ERAD pathway may reflect accumulation of misfolded proteins in the male germline. In corroboration with this assumption, we detected prominent elevation of ubiquitin and ubiquitinated proteins in the knockout spermatozoa (Fig. 5*F*). Collectively, our findings suggest that 1700029I15Rik, in collaboration with the OST complex subunits and folding enzymes, ensures proper biosynthesis of sperm acrosomal membrane glycoproteins in early spermatids (Fig. 5*G*). This working hypothesis is further consolidated by our in vitro observations that 1700029I15Rik promotes the expression of TMEM95 and prevents the formation of a nonnative, SDS-resistant dimer of TMEM95 in vitro (*SI Appendix*, *SI Results and Discussion* and Fig. S10 *A*–*G*).

## Discussion

Nascent polypeptides translocated across the ER membrane usually undergo folding, modifications, and assembly to acquire their native conformations. To maintain protein homeostasis, unfolded or misfolded proteins are eliminated by sophisticated quality control systems (29). In somatic cells, lectin-like ER chaperones CANX and CALR target misfolded proteins for degradation via the ubiquitin-dependent ERAD pathway (29, 30). In the male germline, calmegin (CLGN) and calreticulin-3 (CALR3) are the testis-specific paralogs of CANX and CALR, respectively, and regulate the biosynthesis of sperm plasma membrane proteins (e.g., ADAM1B and ADAM3) required for sperm penetration through the uterotubal junction and ZP binding (31–33). Ablation of CLGN or CALR3 does not impair sperm–egg fusion (33, 34), suggesting that the biosynthesis of gamete fusion-related proteins is regulated by an independent pathway.

Mammalian cells express two distinct forms of OST complexes, centered around catalytic subunits staurosporine and temperature sensitive 3A and 3B (STT3A and STT3B), respectively. STT3A–OST is directly associated with the translocon and cotranslationally regulates *N*-glycosylation of nascent polypeptides, whereas STT3B–OST post-translationally glycosylates fully synthesized proteins (35). 1700029I15Rik interacts with non-catalytic OST subunits DAD1, OSTC, and RPN2; *1700029I15Rik* knockout testes exhibit reduced levels of OSTC and RPN2 (Fig. 4 *A* and *E*). DAD1 and RPN2 are shared accessory subunits of STT3A and STT3B complexes, whereas OSTC exclusively exists in STT3A–OST (28). Previous in vitro studies have demonstrated that depletion of DAD1 destabilizes the OST subunits RPN1, RPN2, and dolichyl-di-phosphooligosaccharide-protein glycotransferase (DDOST) and causes hypoglycosylation (36, 37), whereas ablation of OSTC impairs glycosylation of STT3A-dependent substrates (38). Thus, it is tempting to speculate that loss of 1700029I15Rik might disrupt the structural integrity of the OST complex and potentially hamper the STT3A-mediated cotranslational glycosylation. 1700029I15Rik also interacts with folding chaperones PDIA3 and peptidyl-prolyl cis–trans isomerase B (PPIB), and malectin (MLEC; Fig. 4*A*), a carbohydrate-binding protein involved in the quality control of glycoproteins (39), raising a possibility that 1700029I15Rik has multiple functions in protein processing. In *1700029I15Rik* knockout testes, CD46, EQTN, IZUMO1, and SPACA1 are significantly reduced but remain glycosylated and trafficked to the acrosomal membrane (Fig. 4*F* and *SI Appendix*, Fig. S7*C*). Thus, we hypothesize that deletion of *1700029I15Rik* partially impairs protein glycosylation and disulfide bond formation, resulting in increased protein misfolding; an accumulation of misfolded proteins presumably induces ER stress that transcriptionally upregulates the ERAD machinery. In support of this assumption, we observed elevated ERAD chaperones and ubiquitinated proteins in *1700029I15Rik* knockout sperm (Fig. 5 *C* and *F* and *SI Appendix*, Fig. S9*B*).

CD46, EQTN, IZUMO1, and SPACA1 are downregulated in *1700029I15Rik* knockout testes, but show normal protein levels in the knockout sperm (Fig. 4*F*). The expression of these acrosomal membrane proteins initiates in early round spermatids (*SI Appendix*, Fig. S6*F*). Western blot analyses of acrosomal membrane proteins in whole testis lysates mainly reflect their levels in cytoplasm-rich spermatids, because spermatozoa devoid of cytoplasm only account for a small population in testes. It might be possible that in early spermiogenesis, acrosomal membrane proteins are synthesized at an excess amount in the ER; only a fraction of the proteins is subsequently incorporated into the acrosomal membrane and eventually retained in mature spermatozoa, while the remainder is abandoned with the residual bodies. Therefore, in *1700029I15Rik* knockout males, although the acrosomal membrane proteins show decreased abundance in testes relative to the wild-type, the amounts are sufficient to secure endogenous protein levels in mature spermatozoa. This hypothesis might explain the discrepancies in the protein levels observed in the immunoblots of testis and sperm samples but requires experimental verification.

Proteins destined for sperm plasma membrane and acrosomal membrane exhibit striking differences in the timing of mRNA expression. For example, sperm plasma membrane proteins, ADAM1B and ADAM3, show peak expression in late spermatocytes, whereas acrosomal membrane proteins, IZUMO1 and SPACA1, exhibit biased expression in mid-round spermatids (*SI Appendix*, Fig. S6*F*). Consistent to the expression timing of their putative substrates, CLGN and 1700029I15Rik are highly expressed in late spermatocytes and early spermatids, respectively (*SI Appendix*, Figs. S1*B* and S6*F*). Thus, we propose that the biosynthesis pathways of sperm plasma membrane and acrosomal membrane proteins are independently regulated in a spatiotemporal manner (*SI Appendix*, Fig. S11). However, further investigation is warranted to determine whether the substrates of 1700029I15Rik are restricted to acrosomal membrane glycoproteins.

1700029I15Rik, which supposedly functions in the ER, is initially detected in the acrosomal granule of round spermatids and becomes absent in elongating spermatids (*SI Appendix*, Fig. S4*A*). Early studies have reported that the ER chaperone CALR also localizes to the acrosomal granule of mouse and rat spermatids (40, 41). Similarly, the ER-resident protein OSTC first appears as a granule-like structure in spermatocytes, subsequently relocates to the acrosomal granule in round spermatids, and is eventually discarded via shedding of residual bodies (*SI Appendix*, Fig. S6*B*). This disposal mechanism of ER-resident proteins may explain how 1700029I15Rik becomes absent in elongating spermatids. Future studies are necessary to explore whether 1700029I15Rik has a function in the acrosomal granule.

Recently, two independent studies on *1700029I15Rik* were published (42, 43). Despite that the knockout mouse lines exhibit the same phenotype, the works by three groups show great discrepancies in terms of the proposed molecular functions of 1700029I15Rik. Contreras et al. (42) discovered that 1700029I15Rik retains IZUMO1 in the ER of HeLa cells, contradicting with our observations that IZUMO1 colocalizes with 1700029I15Rik at the surface of COS-7 cells (*SI Appendix*, Fig. S1*I*) and that coexpression with 1700029I15Rik does not prominently alter the subcellular expression pattern of IZUMO1 in HEK293T cells (*SI Appendix*, Fig. S10*H*). We reason that the use of different cell lines may account for the different localization of ectopically expressed 1700029I15Rik. By immunohistochemistry, Contreras et al. observed that IZUMO1 shows reduced colocalization with BIP (also known as HSPA5) in the acrosomal granule of knockout spermatids as compared to the wild-type (42). We argue that additional evidence is required to support the conclusion that 1700029I15Rik mediates ER retention of IZUMO1 in vivo. More importantly, IZUMO1 is an acrosomal membrane protein that does not localize to the acrosomal granule of wild-type spermatids. We reason that the fluorescence signals detected in the proacrosome might be affected by the angle of sectional plane during imaging (e.g., *Lower Right* panel of Fig. 3*H*). Based on a previous discovery of an IZUMO1-containing multiprotein complex (44), the authors proposed a working model that 1700029I15Rik transiently retains IZUMO1 in the ER, thereby allowing IZUMO1 to coassemble with other fertilization-related proteins (42). However, it remains open questions whether this high-molecular-weight protein complex is assembled in the ER, and whether the aberrant assembly of this complex would negatively affect sperm–egg binding or fusion.

Distinct to the observations made by Contreras et al. (42) and our study (*SI Appendix*, Fig. S2*J*), Hao et al. (43) reported that *1700029I15Rik* knockout male mice show significantly reduced sperm counts. The authors performed proteomic analyses on wild-type and knockout whole testis and sperm and identified a multitude of downregulated proteins in the knockout groups (43). We are concerned that the decreased sperm counts in that mouse line may reflect changes in the germ cell populations (i.e., reduced percentages of round spermatids or testicular sperm in knockout testes), which potentially complicates the interpretation of the MS outcomes. Different from our Western blot outcomes (Fig. 4*H*), the proteomic analysis by Hao et al. revealed that IZUMO1 and EQTN are downregulated in the knockout sperm (43).

It is worth noting that gamete fusion-related acrosomal membrane proteins, such as TMEM95 and SPACA6, had not been detected in any of the testis or sperm proteomic analyses in the present study (Dataset S1). This is likely due to their extremely low levels of expression, which also hinder us from visualizing their subcellular localization by immunohistochemistry. These proteins might be effectively enriched in round spermatids isolated from testes; however, the velocity sedimentation (STA-PUT) or fluorescence-activated cell sorting (FACS)-based germ cell purification involves protease treatments and prolonged exposure to an in vitro environment, thus potentially introducing unexpected alterations in the proteome. To comprehensively elucidate the characteristics of such low-abundance proteins, we envision that immunostaining with signal amplification by exchange reaction (Immuno-SABER) and proximity-dependent biotinylation identification (BioID) could be employed in future investigations (45, 46).

In summary, this study suggests that 1700029I15Rik specifically regulates the biosynthesis of acrosomal membrane proteins by ensuring precise and timely coordination of multiple protein modification events. 1700029I15Rik interacts with proteins involved in *N*-glycosylation, disulfide isomerization, and vesicular trafficking. Ablation of *1700029I15Rik* destabilizes the OST subunits and likely disrupts *N*-glycosylation of acrosomal membrane proteins. The aberrantly glycosylated or improperly disulfide-bonded proteins are misfolded and eliminated by the ubiquitin-dependent ERAD pathway, thereby resulting in the reduced abundance or absence of mature proteins (Fig. 5*G*). This pathway acts independently to the CLGN-mediated processing of sperm plasma membrane proteins (*SI Appendix*, Fig. S11). Given that 1700029I15Rik is highly conserved in humans, this work may open avenues for the development of a nonhormonal contraceptive targeting the biosynthesis of gamete fusion-required acrosomal proteins during spermiogenesis.

## Materials and Methods

For immunodetection of 1700029I15Rik, a polyclonal antibody against its C terminus was raised in rabbits. The mutant mouse lines lacking *1700029I15Rik* were generated by CRISPR/Cas9. Fertility, histological, sperm motility, and in vitro fertilization analyses were carried out to investigate the phenotype of knockout males. Co-IP/MS analyses were conducted to identify the interacting proteins of 1700029I15Rik in mouse testes. Proteomic changes in *1700029I15Rik* knockout testes and sperm were analyzed by MS and Western blotting. In vitro analyses were performed using HEK293T and COS-7 cells to elucidate the protein features and molecular functions of 1700029I15Rik. Detailed experimental procedures are provided in *SI Appendix*, *SI Materials and Methods*.

## Supplementary Material

Appendix 01 (PDF)

Dataset S01 (XLSX)

## Data Availability

Mass spectrometry raw data have been deposited in ProteomeXchange Consortium via the Proteomics Identification Database (PRIDE) with the dataset identifier (47). All study data are included in the article and/or *SI Appendix*.
