# Supplementary material for: 1700029I15Rik orchestrates the biosynthesis of acrosomal membrane proteins required for sperm–egg interaction
Source: Proc Natl Acad Sci U S A. 2023 Feb 14;120(8):e2207263120. doi: 10.1073/pnas.2207263120 (PMC9974436; doi:10.1073/pnas.2207263120)
Supplement: Supplementary file 1 — Appendix 01 (PDF) [file pnas.2207263120.sapp.pdf]

Supporting Information for

1700029I15Rik orchestrates the biosynthesis of acrosomal membrane proteins required for sperm–egg interaction

Yonggang Lu, Kentaro Shimada, Shaogeng Tang, Jingjing Zhang, Yo Ogawa, Taichi Noda, Hiroki Shibuya, Masahito Ikawa

Corresponding author: Masahito Ikawa

Email: ikawa@biken.osaka-u.ac.jp

This PDF file includes:

SI Materials and Methods

SI Results and Discussion

Tables S1 to S3

Figures S1 to S11

Legend for Dataset S1

SI References

Other supporting materials for this manuscript include the following:

Dataset S1

SI Appendix

SI Materials and Methods

Animals

C57BL/6J, B6D2F1, ICR and SWISS mice were purchased from Japan SLC, Inc. (Shizuoka, Japan) or Janvier Labs (Le Genest Saint Isle, France) and maintained under cycles of 12 h light and 12 h darkness with *ad libitum* feeding. All animal experiments were approved by Institutional Animal Care and Use Committees at the Research Institute for Microbial Diseases, Osaka University (#Biken-AP-H30-01) and the University of Gothenburg (#1316/18), and were conducted in compliance with all guidelines and regulations. Mice were euthanized by cervical dislocation following anesthesia.

Frozen spermatozoa from *1700029I15Rik* heterozygous knockout males (B6D2-1700029I15Rik<em1Osb>, RBRC#11049, CARD#2956), and *1700029I15Rik* transgenic (Tg) males (B6D2-Tg(Clgn-1700029I15Rik/PA)1Osb, RBRC#11470, CARD#3114) are available at the Riken BioResource Center (RIKEN BRC; web.brc.riken.jp/en) and the Center for Animal Resources and Development, Kumamoto University (CARD R-BASE; cardb.cc.kumamoto-u.ac.jp/transgenic).

Reverse transcription polymerase chain reaction (RT-PCR)

Total RNA was extracted from various tissues and organs of C57BL/6J mice by TRIzol (ThermoFisher, Waltham, MA) and reverse transcribed to cDNA using the SuperScript III First Strand Synthesis Kit (ThermoFisher, Waltham, MA) as per manufacturer's instruction. The mRNA expression was then analyzed by PCR using the KOD Fx Neo DNA Polymerase (Toyobo, Tokyo, Japan). Sequences of the primers for RT-PCR are listed in the ***SI Appendix*, Table S3**.

Phylogenetic and taxonomic analyses

The protein sequence of murine 1700029I15Rik was input as a query sequence for a protein BLAST (Basic Local Alignment Search Tool) search using the blastp (protein–protein BLAST) algorithm. 33 protein sequences from species of Euarchontoglires and 13 from species of Laurasiatheria were selected for phylogenetic tree visualization by iTOL (1). Taxonomic analysis was performed using the NCBI Taxonomy CommonTree tool (2).

Evolutionary conservation of protein residues by ConSurf

The protein sequence of murine 1700029I15Rik was input as a query sequence for a protein BLAST search using the blastp algorithm. The top 150 results were filtered manually and ortholog-unique sequences were subjected to alignment by MAFFT (3). The multiple sequence alignment and the structure of 1700029I15Rik predicted by AlphaFold (4) were used as input in the ConSurf server (5).

In silico analysis of gene expression in spermatogenic cells

The mRNA expression of mouse *1700029I15Rik*, *Adam1b*, *Adam3*, *Canx*, *Cd46*, *Dcst1*, *Dcst2*, *Eqtn*, *Fimp*, *Izumo1*, *Sof1*, *Spaca1*, *Spaca4*, *Spaca6*, *Tmem95*, and *Zp3r* in male germ cells at different spermatogenic stages was evaluated based on the scRNA-seq dataset published by Hermann et al. (6).

Antibodies and plasmids

Antibodies used in this study are listed in ***SI Appendix*, Table S2**. Polyclonal antibodies against aa 81 – 99 of mouse 1700029I15Rik

(QQRKRDGPNMADYYYDVNL) and aa 75 – 93 of mouse SPACA6 (EGAFEDLKDMKINYDERSYL) were raised in rabbits and produced by Sigma-Aldrich (Sigma-Aldrich, St. Louis, MO). Briefly, rabbits were immunized with synthetic peptides containing an N-terminal cysteine residue. Polyclonal antibodies were purified from the rabbit antisera with synthetic peptide-conjugated SulfoLink™ Coupling Resin (ThermoFisher, Waltham, MA) according to the manufacturer's instruction.

The open reading frames (ORFs) of *1700029I15Rik*, *Izumo1*, *Spaca6*, and *Tmem95* were cloned and amplified from mouse or human testis cDNA by PCR and inserted into the multiple cloning site of pCAG1.1 vector (Addgene; Plasmid #173685).

Triton X-114 fractionation of testis and sperm proteins

Triton X-114 (Sigma-Aldrich, St. Louis, MO) was precondensed as previously described (7). Testes were homogenized on ice in 1% Triton X-114 lysis buffer [1% (v/v) Triton X-114 and 1% (v/v) protease inhibitor cocktail (Nacalai Tesque, Kyoto, Japan) in phosphate buffered saline (PBS)] using a Dounce homogenizer. Cauda epididymal sperm were lysed in the same buffer at 4 °C for 2 h with occasional vortexing. The insoluble cell debris were removed by centrifugation at 15,000 × *g*, 4 °C for 30 min. The cleared testis or sperm lysates were then incubated at 37 °C for 15 min and centrifuged at 15,000 × *g*, room temperature for 30 min. The upper aqueous phase containing soluble proteins was transferred to a fresh conical tube. To remove impurities from both fractions, the aqueous phase was mixed with precondensed Triton X-114 at a dilution of 1:100 and the detergent phase was mixed with 100 volumes of PBS. The solutions were fractionated again by incubation at

37 °C and centrifugation at 15,000 × *g*, room temperature; this purification step was repeated for three times. The purified detergent phase was diluted with PBS to the volume of the aqueous phase. An equal volume of both fractions was denatured in sodium dodecyl sulfate (SDS) sample buffer [66.7 mM Tris-HCl (pH 6.8), 2% (w/v) SDS, 0.003% (w/v) bromophenol blue, 10% (v/v) Glycerol, and 5% β-mercaptoethanol in distilled water] at 95 °C for 10 min, resolved by sodium dodecyl sulfate-polyacrylamide gel electrophoresis (SDS-PAGE), and analyzed by western blotting.

Protein extraction and Western blotting

HEK293T cells were lysed in 1% Triton X-100 lysis buffer [1% Triton X-100, 50 mM Tris-HCl (pH 7.5), 150 mM NaCl, 10% glycerol, and 1% (v/v) protease inhibitor cocktail (Nacalai Tesque, Kyoto, Japan) in distilled water] at 4 °C for 2 h with occasional vortexing. Mouse testes were decapsulated with micro spring scissors and fine forceps under a dissection microscope and were homogenized in 1% Triton X-100 lysis buffer using a Dounce homogenizer. Mature sperm were extracted from mouse cauda epididymides and suspended in 1 mL PBS by gentle pipetting. After three washes in PBS, the sperm concentrations were determined using a hemocytometer. Each sperm sample was mixed with an appropriate volume of radioimmunoprecipitation (RIPA) buffer [1% Triton X-100, 50 mM Tris-HCl (pH 7.5), 150 mM NaCl, 0.5% (w/v) sodium deoxycholate, 0.1% (w/v) SDS, and 1% protease inhibitor cocktail in distilled water] to make a final concentration of 1.6×10^8 sperm/mL, and lysed at 4 °C for 2 h with occasional vortexing. HEK93T cell, testis, and sperm lysates were cleared by centrifugation at 20,000 × *g*, 4 °C for 15 min. The supernatants were transferred to fresh conical tubes and the protein concentrations

of HEK293T cell or testis lysates were measured by Bradford protein assay on a Nanodrop™ spectrophotometer (Thermofisher, Waltham, MA). The protein concentration of sperm lysates was not measured. Instead, an equal volume of wildtype and knockout sperm lysates was loaded for subsequent SDS-PAGE.

For Western blot analyses carried out under reducing and denaturing conditions, protein lysates were boiled at 95 °C for 10 min in the SDS sample buffer containing β-mercaptoethanol. For the samples under non-denaturing and non-denaturing conditions, the lysates were mixed with the SDS sample buffer without β-mercaptoethanol and directly resolved on SDS-PAGE. Protein lysates were loaded on 5 – 20% SDS-PAGE gradient gels (e-PAGEL, ATTO Corp., Osaka, Japan) and transferred to polyvinylidene fluoride (PVDF) membranes using the Trans-Blot Turbo system (BioRad, Munich, Germany). After blocking with 10% Difco™ skim milk (BD Bioscience, Sparks, MD) in Tris-buffered saline containing 0.1% Tween 20 (TBST; Nacalai Tesque, Kyoto, Japan), the PVDF membranes were incubated with primary antibodies for 3 h at room temperature or overnight at 4 °C and washed in TBST for three times. The membranes were then probed with horseradish peroxidase (HRP)-conjugated secondary antibodies (Jackson ImmunoResearch Laboratories, West Grove, PA) for 1 h at room temperature, followed by additional three washes in TBST. Finally, the membranes were immersed in Amersham™ ECL™ Western Blotting Reagent (Cytiva, Tokyo, Japan) for 15 s and protein bands were detected by Amersham™ ImageQuant™ 800 (Cytiva, Tokyo, Japan).

The anti-SPACA6 polyclonal antibody is highly sensitive to mouse IgG contamination. For Western blot detection of SPACA6 in testis lysates, the testes were decapsulated under a dissection microscope and lysed in 1% Triton X-114. The detergent fractions were isolated and washed as described above, denatured in SDS

sample buffer containing β-mercaptoethanol, and subjected to SDS-PAGE and Western blot analyses. For immunodetection of sperm SPACA6, cauda epididymal sperm were washed twice in PBS. Blood cells in the sperm samples were hemolyzed by exposing the cells to distilled water, a hypotonic environment, for 1 min. The sperm solutions were immediately adjusted to isotonicity by adding 1/10 volumes of 10×PBS (Nacalai Tesque, Kyoto, Japan). The sperm cells were washed in PBS twice more and subjected to protein extraction in RIPA buffer.

Transfection and immunostaining of HEK293T and COS-7 cells

HEK293T and COS-7 cells were maintained in Dulbecco's Modified Eagle Medium (DMEM) complete medium [DMEM (Thermofisher, Waltham, MA) supplemented with 10% fetal bovine serum (Sigma-Aldrich, St. Louis, MO) and 1% Gibco™ penicillin/streptomycin (Thermofisher, Waltham, MA)] at 37 °C, 5% CO_2 and subcultured every three to four days. Plasmids encoding proteins of interest were introduced into HEK293T or COS-7 cells by calcium phosphate–DNA co-precipitation or using Polyethylenimine Max (Polysciences, Inc., Warrington, PA), respectively. Culture media containing transfection reagents were replaced with prewarmed DMEM complete media at 12 h post transfection.

For immunocytochemistry analyses, HEK293T and COS-7 cells were seeded on 0.001% poly-L-lysine (Sigma-Aldrich, St. Louis, MO)-coated round coverslips (Matsunami Glass Co. Ltd., Osaka, Japan). For live cell immunostaining, DMEM complete medium was sterile filtered through a 0.2-μm disk filter (Kurabo, Osaka, Japan) and prewarmed to 37 °C. The entire immunostaining procedure was performed at 37 °C, 5% CO_2 until microscopic imaging. Briefly, cells on coverslips were washed in prewarmed DMEM medium three times and probed by primary

antibodies in the culture medium for 1 h. After three washes in prewarmed medium, the cells were then incubated in the medium containing fluorophore-conjugated secondary antibodies another 1 h. After another three washes, cells were stained with CellMask™ deep red plasma membrane stain (Thermofisher, Waltham, MA) and Hoechst 33342 (Thermofisher, Waltham, MA) in the DMEM medium for 10 min, followed by three washes in fresh medium. The coverslips were mounted in a drop of filtered DMEM medium, with the cells facing towards the microscope slide. The cells were imaged immediately under a Nikon Eclipse Ti microscope equipped with a Nikon C2 confocal module (Nikon, Tokyo, Japan).

Alternatively, immunocytochemistry was performed on fixed cells at room temperature. After three washes in PBS, cells were fixed in 4% paraformaldehyde in PBS (Thermofisher, Waltham, MA) for 10 min at room temperature. The fixed cells were washed three times in PBS, followed by permeabilization in 0.1% Triton X-100 in PBS for 1 h. After incubating the cells in blocking buffer [0.1% Triton X-100, 10% Gibco™ goat serum (Thermofisher, Waltham, MA), and 3% bovine serum albumin (Sigma-Aldrich, St. Louis, MO) in PBS] for 1 h, the cells were probed with primary antibodies in blocking buffer for 3 h, followed by three washes in 0.1% Triton X-100 in PBS. The cells were then incubated with fluorophore-conjugated secondary antibodies in dark for 1 h, followed by three washes in 0.1% Triton X-100 in PBS and additional three washes in PBS. The cells were finally stained with CellMask™ deep red plasma membrane stain and Hoechst 33342 in PBS for 10 min and washed in PBS for three times, followed by an additional wash in distilled water. The coverslips were mounted in a drop of VECTASHIELD® Antifade Mounting Medium (Vector Laboratories, Tokyo, Japan), with the cells facing towards the microscope slide. The

cells were imaged under a Nikon Eclipse Ti microscope equipped with a Nikon C2 confocal module (Nikon, Tokyo, Japan).

Proteinase K protection assay

HEK293T cells were transfected by calcium phosphate–DNA co-precipitation and the culture medium was replaced 12 h after transfection. After 24 h of transfection, the culture medium was aspirated to remove detached HEK293T cells and replaced with prewarmed DMEM complete medium. The cells were then incubated with 0.1 mg/mL of proteinase K in DMEM complete medium at 37 °C, 5% CO_2 for 20 min. The enzyme-treated cells were immediately transferred to conical tubes and placed on ice. Cells were washed three times by centrifuging at 1,000 × *g*, 4 °C for 5 min and resuspending in ice-cold PBS, and were lysed in 1% Triton X-100 lysis buffer containing 1% protease inhibitor cocktail for Western blot analyses.

Generation of *170029I15Rik* knockout, mutant, and transgenic mouse lines

1700029I15Rik knockout mice (*1700029I15Rik*$^{+/-}$) were generated by the CRIPSR/Cas9-based gene editing technology as previously described (8). Two sgRNAs targeting the first coding exon and the 3' UTR of *1700029I15Rik* were designed to remove the entire coding sequence. Wildtype B6D2F1 female mice were superovulated by peritoneal injection of CARD HyperOva (Kyudo Co., Saga, Japan) and human chorionic gonadotropin (hCG; ASKA Pharmaceutical Co. Ltd., Tokyo, Japan) and paired with B6D2F1 males. Two-pronuclear (2PN) zygotes were collected from the B6D2F1 female mice. A ribonucleoprotein complex of CRISPR RNA (crRNA), trans-activating crRNA (tracrRNA), and Cas9 protein was introduced into the 2PN eggs using a NEPA21 super electroporator (NEPA GENE, Chiba,

Japan). The electroporated zygotes were cultured in the potassium simplex optimisation medium (KSOM) to two-cell stage and transplanted into the ampullary segment of the oviducts of 0.5-d pseudopregnant ICR females. The founder generation was obtained by natural delivery or Cesarean section and genotyped by PCR using primers (Fw and Rv) targeting the introns upstream and downstream of the coding region. The mutant allele was subsequently examined by Sanger sequencing.

Similarly, the *1700029I15Rik* mutant mouse line (*1700029I15Rik*$^{+/\Delta}$) was generated using two sgRNAs targeting the upstream and downstream introns of the first coding exon. Mouse zygotes were obtained from mating between C57BL/6J females and males. Cas9 protein (Sigma-Aldrich, St. Louis, MO) and sgRNAs were mixed to form ribonucleoprotein complexes and co-injected into the zygotes in the M2 medium. Microinjected zygotes were allowed to recover for 2 – 4 h in the KSOM medium in a humidified CO_2 incubator at 37 °C and were then transferred into pseudopregnant SWISS female mice. Likewise, the founders were genotyped using primers (Fw' and Rv') flanking the mutation site and the PCR products were subjected to Sanger sequencing. The founder mice were crossed with C57BL/6J mice to avoid potential off-target mutations.

For producing the transgenic mouse line, the ORF of *1700029I15Rik* was cloned from mouse testis cDNA by PCR and inserted into a plasmid encoding a *Clgn* promoter and a rabbit beta globin polyadenylation (polyA) signal. Linearized plasmids were microinjected into the pronuclei of zygotes obtained from mating between homozygous knockout females and heterozygous knockout males. The injected zygotes were cultured in the KSOM medium until the two-cell stage and transplanted into the oviductal ampulla of 0.5 d pseudopregnant ICR females.

Founder animals were obtained by natural delivery or Cesarean section after 19 d of pregnancy. The transgene was identified by PCR using primers (Tg_Fw and Tg_Rv) targeting the *Clgn* promoter and the polyA signal.

The primers used for genotyping the knockout, mutant, and transgenic mice are enumerated in ***SI Appendix*, Table S3**.

Fertility tests

Upon sexual maturation, homozygous knockout male mice or knockout Tg males were caged individually with three 6-week-old wildtype B6D2F1/J female mice for eight weeks. During this period, vaginal plugs was examined as an indicator of successful copulation and the number of pups was counted at birth. Three knockout males were tested to meet the requirements for statistical validity. The fecundity of three wildtype B6D2F1/J males was tested in parallel as positive controls. After the eight-week mating period, the male mice were withdrawn from the cages and the females were kept for another three weeks to allow their final litters being delivered. For the fertility tests of *1700029I15Rik* mutant mice, one male was housed with one female mouse consecutively for six months. The number of offspring was recorded at birth.

Histological analyses of testes and epididymides

Testes and epididymides were fixed in Bouin's fluid (Polysciences, Inc., Warrington, PA), embedded in paraffin wax, and sectioned at a thickness of 5 – 10 μm on a Microm HM325 microtome (Microm, Walldorf, Germany). The paraffin sections were stained with periodic acid (Nacalai Tesque, Kyoto, Japan) and Schiff's reagent (Wako, Osaka, Japan), followed by counterstaining with Mayer's hematoxylin

solution (Wako, Osaka, Japan). Alternatively, the testis sections were stained with hematoxylin and eosin.

Immunostaining of spermatocyte chromosome spreads

Testicular germ cells were extracted from wildtype and mutant testes, washed in PBS, centrifuged, and resuspended in hypotonic buffer [30 mM Tris-HCl (pH 7.5), 17 mM trisodium citrate, 5 mM EDTA, and 50 mM sucrose], followed by centrifugation and resuspension in 100 mM sucrose. The cell suspensions were then smeared on microscope slides in a same volume of fixation buffer (1% paraformaldehyde and 0.1% Triton X-100), fixed at room temperature for 3 h, and air dried. For immunostaining, the testicular germ cells were incubated with primary antibodies in PBS containing 5% BSA for 2 h, followed by staining with fluorophore-conjugated secondary antibodies for 1 h at room temperature. The cells were washed with PBS and mounted in VECTASHIELD® Antifade Mounting Medium with DAPI (Vector Laboratories, Burlingame, CA) prior to imaging.

***In vitro* fertilization**

Cauda epididymal spermatozoa extracted from sexually mature wildtype and knockout males were preincubated in the TYH medium for 2 h at 37 °C, 5% CO_2 to induce capacitation. Cumulus-oocyte complexes (COCs) were extracted from the oviductal ampulla of superovulated B6D2F1 female mice, and were treated with 330 μg/mL of hyaluronidase (Wako, Osaka, Japan) to disperse the cumulus cells or 1 mg/mL of collagenase (Sigma-Aldrich, St. Louis, MO) to remove both the cumulus cells and the ZP. Cumulus-intact and cumulus-free eggs were incubated with spermatozoa at a concentration of 2×10^5 sperm/mL in 100 μL TYH medium drops,

whereas ZP-free eggs were inseminated at a density of 2 × 10^4 sperm/mL. After 6 h of incubation, fertilization success was determined by the formation of two pronuclei.

***In vivo* fertility test**

B6D2F1 female mice were superovulated by peritoneal injection of PMSG and hCG and paired with wildtype or *1700029I15Rik*$^{-/-}$ males. Oviducts were isolated from the female mice 6 h after the formation of copulation plugs. COCs extracted from the oviductal ampulla were incubated in the KSOM medium and treated with 330 μg/mL of hyaluronidase (Wako, Osaka, Japan) to remove the cumulus cells. The eggs were then fixed on ice in FHM medium containing 0.25% (v/v) glutaraldehyde (Polysciences, Inc., Warrington, PA) for 15 min, washed in fresh FHM medium for three times, and stained with Hoechst 33342 at room temperature for 15 min to visualize pronuclei and perivitelline sperm. The numbers of perivitelline sperm were counted and Z-stack images were captured under a Nikon Eclipse Ti microscope equipped with a Nikon C2 confocal module (Nikon, Tokyo, Japan).

Sperm–egg binding and fusion assay

For analyzing the oolemma binding ability, cauda epididymal spermatozoa were preincubated in TYH medium at 37 °C, 5% CO_2 for 2 h. To visualize the acrosomal status, the capacitated spermatozoa were probed with an anti-EQTN antibody and an Alexa Fluor™ 488-conjugated secondary antibody in a TYH medium drop. Meanwhile, the COCs were extracted from superovulated females and treated with 1 mg/mL of collagenase to remove both the cumulus cells and the ZP. The eggs were then incubated with the antibody-probed spermatozoa at a density of 2 × 10^4 sperm/mL. After insemination for 30 min, the sperm–egg complexes were fixed on

ice in FHM medium drops containing 0.25% glutaraldehyde for 15 min, gently washed in fresh FHM medium for three times, and stained with Hoechst 33342 to visualize the bound sperm. The total number of sperm bound to the oolemma and the number of acrosome-reacted sperm bound to the oolemma were counted under a Keyence BZ-X810 microscope (Keyence, Osaka, Japan). Z-stack images were captured under a Nikon Eclipse Ti microscope equipped with a Nikon C2 confocal module (Nikon, Tokyo, Japan).

Sperm–egg fusion assay was performed as previously described (9). Briefly, ZP-free eggs were stained with Hoechst 33342 for 15 min at a dilution of 1:10,000, washed thoroughly in fresh FHM medium drops for six times, and incubated with capacitated sperm in TYH medium. After 30 min of insemination, the sperm–egg complexes were fixed in 0.25% glutaraldehyde in FHM medium, washed in fresh FHM medium for three times, and imaged under a Keyence BZ-X810 microscope (Keyence, Osaka, Japan).

Analyses of sperm count, morphology, and motility

Spermatozoa were extracted from cauda epididymides of adult male mice under a dissection microscope using micro spring scissors and fine forceps. For sperm count and morphology analyses, sperm were suspended in 1 mL PBS by gentle pipetting. Sperm density was measured using a hemocytometer and sperm morphology was observed under an Olympus BX-53 differential interference contrast microscope equipped with an Olympus DP74 color camera (Olympus, Tokyo, Japan). For motility analysis, sperm were dispersed in 100 µL of TYH medium drops. Sperm motility was measured by the CEROS II sperm analysis system (Hamilton Thorne Biosciences, Beverly, MA) at 10 min and 2 h of incubation in TYH medium.

Sperm ZP binding assay

Cauda epididymal sperm were pre-incubated in TYH medium for 2 h at 37 °C, 5% CO_2. The eggs harvested from B6D2F1 female mice were denuded by treating with 330 μg/mL hyaluronidase at 37 °C for 5 min. The capacitated sperm were added to TYH medium drops containing cumulus-free eggs at a final density of 2 × 10^5 sperm/mL and incubated at 37 °C, 5% CO_2 for 30 min. The sperm–egg complexes were fixed in 0.25% glutaraldehyde for 15 min on ice, rinsed in fresh FHM medium drops for three times. The numbers of sperm bound to the ZP were counted and the eggs were captured under an Olympus BX-53 differential interference contrast microscope equipped with an Olympus DP74 color camera (Olympus, Tokyo, Japan).

Immunostaining of testis cryosections and spermatozoa

Testes were fixed in 4% paraformaldehyde in PBS at 4 °C for overnight, washed three times in PBS, and incubated in 15% and subsequently 30% sucrose in PBS at 4 °C until the testes sank. The testes were then embedded in the Tissue-Tek® optimal cutting temperature (O.C.T.) compound (Sakura Finetek USA, Inc., Torrance, CA) and snap-frozen in liquid nitrogen. Testis blocks were sectioned at a thickness of 5 – 10 μm on a CryoStar NX70 cryostat (ThermoFisher, Waltham, MA). The testis sections were dried on adhesive microscope slides at 37 °C for 15 min (Matsunami Glass Co. Ltd., Osaka, Japan), permeabilized in 0.1% Triton X-100 in PBS at room temperature for 1 h, and incubated with blocking buffer [0.1% Triton X-100, 10% Gibco™ goat serum (Thermofisher, Waltham, MA), and 3% bovine serum albumin (Sigma-Aldrich, St. Louis, MO) in PBS] at room temperature for another 1 h. The sections were then probed with primary antibodies for 3 h at room temperature or

overnight at 4 °C. After three washes in 0.1% Triton X-100 in PBS, the sections were incubated with fluorophore-conjugated secondary antibodies for 1 h at room temperature, washed with 0.1% Triton X-100 in PBS for three times, and then stained with Hoechst 33342 (ThermoFisher, Waltham, MA) for 30 min at room temperature. After additional three washes in PBS, the sections were mounted with Epredia™ Immu-Mount (Fisher Scientific, Pittsburgh, PA) prior to imaging under a Nikon Eclipse Ti microscope equipped with a Nikon C2 confocal module (Nikon, Tokyo, Japan).

Spermatozoa extracted from cauda epididymides were air dried on microscope slides at 37 °C and fixed in 4% paraformaldehyde in PBS for 30 min at room temperature. The fixed sperm were then processed in the same manner as immunostaining of testis cryosection. The spermatozoa were mounted with VECTASHIELD® Antifade Mounting Medium (Vector Laboratories, Burlingame, CA) and fluorescence images were captured under a Nikon confocal microscope.

HEK293T cell–egg binding assay

HEK293T cells were transfected with plasmids encoding C-terminal 3 × FLAG-tagged 1700029I15Rik or mCherry-tagged IZUMO1 by the calcium phosphate–DNA co-precipitation method. The culture media containing transfection reagents were replaced with fresh complete DMEM media at 12 h post transfection. After 18 h of transfection, HEK293T cells were detached from the culture dish by gentle pipetting, washed twice in PBS, and resuspended in the TYH medium. ZP-free eggs were incubated with 1×10^4 HEK293T cells in 100 µL TYH medium drops at 37 °C, 5% CO_2 for 30 min. The eggs were washed briefly in fresh TYH medium drops to remove loosely bound cells and imaged under a Keyence microscope.

Co-immunoprecipitation

Testes were homogenized on ice in 1% Triton X-100 lysis buffer containing 1% protease inhibitor cocktail (Nacalai Tesque, Kyoto, Japan) using a Dounce homogenizer. The testis lysates were cleared by centrifugation at 15,000 × *g*, 4 °C for 30 min. The supernatants were transferred to fresh conical tubes and kept on ice. Co-IP was performed using the Invitrogen™ Dynabeads™ Magnetic Beads or Pierce™ Crosslink IP Kit (ThermoFisher, Waltham, MA) in accordance with the manufacturer's instructions.

Silver staining

The protein samples were mixed with the SDS sample buffer, boiled at 95 °C for 10 min, and resolved by SDS-PAGE. The gel was stained using Sil-Best Stain One (Nacalai Tesque, Kyoto, Japan) or Sil-Best Stain-Neo (Nacalai Tesque, Kyoto, Japan) according to the manufacturer's instructions.

Mass spectrometry

Protein samples immunoprecipitated from testis lysates or extracted from mature spermatozoa were processed and the resultant protein peptides were subjected to nanocapillary reversed-phase liquid chromatography (LC)-MS/MS analysis using a C18 column (10 cm × 75 µm, 1.9 µm, Bruker Daltonics) on a nanoLC system (Bruker Daltonics, Billerica, MA) connected to a timsTOF Pro mass spectrometer (Bruker Daltonics, Billerica, MA) and a nano-electrospray ion source (CaptiveSpray; Bruker Daltonics, Billerica, MA). The resulting data were processed using DataAnalysis (Bruker Daltonics, Billerica, MA), and proteins were identified using MASCOT Sever

(Matrix Science, Tokyo, Japan) compared with the SwissProt database. Quantitative value and fold exchange were calculated by Scaffold 5 software (Proteome Software, Portland, OR). All MS results are included in **Dataset S1**.

Statistical analyses

Data are presented as mean values and error bars indicate standard deviation (SD). Experimental groups were analysed statistically using an unpaired two-tailed Student's *t*-test. *P* values less than 0.05 were considered statistically significant (*, $P < 0.05$; **, $P < 0.01$; ***, $P < 0.001$).

Data availability

The raw data of MS analyses have been deposited to the ProteomeXchange Consortium via the PRIDE partner repository (10) with the dataset identifier PXD033246.

SI Results and Discussion

1700029I15Rik is an ER-localizing protein facilitating the biosynthesis of acrosomal membrane proteins

We carried out a series of *in vitro* analyses to further explore the molecular functions of 1700029I15Rik. In line with the discovery that 1700029I15Rik interacts with multiple ER proteins, we observed that this protein is co-localized with KDEL, an ER marker, in HEK293T and COS-7 cells (***SI Appendix*, Fig. S9 *C*** and ***D***). Murine TMEM95 contains two *N*-linked glycosylation sites, N36 and N118 (**SI Appendix, Fig. S10*A***). Overexpression of 1700029I15Rik in HEK293T cells significantly enhanced the expression of non-glycosylated TMEM95 at 24 h post transfection (***SI Appendix*, Fig. S10 *B*** and ***C***). Distinctly, 1700029I15Rik did not improve the expression of IZUMO1 or SPACA6, which are efficiently and extensively glycosylated in HEK293T cells (***SI Appendix*, Fig. S10 *D*** and ***E***). Proteinase K treatment revealed no significant change in the surface expression of TMEM95 with or without the presence of 1700029I15Rik (***SI Appendix*, Fig. S10 *B*** and ***F***). Further, co-expression of 1700029I15Rik inhibited the dimerization of fully glycosylated TMEM95 at 36 h after transfection (***SI Appendix*, Fig. S10*G***). Since TMEM95 exists as an SDS-resistant monomer in human spermatozoa (11), we conjecture that the formation of the non-native, SDS-resistant dimer of TMEM95 might be a consequence of incorrect protein folding without the presence of 1700029I15Rik.

As revealed by co-IP/MS, 1700029I15Rik potentially interacts with ER–Golgi vesicular trafficking protein, including COPI coat complex subunit gamma 1 (COPG1), TMED4, and TMED10 (**Fig. 4*A***). These protein families have been reported to be implicated in protein trafficking during acrosome biogenesis (12, 13). However, overexpression of 1700029I15Rik did not alter the extent of surface

expression of TMEM95 or IZUMO1 in HEK293T cells (***SI Appendix*****, Fig. S10** ***B*** and ***H***), suggesting that 1700029I15Rik might not have a role in protein transport. Alternatively, it might be possible that the vesicular trafficking proteins interacts with and target 1700029I15Rik to the acrosomal granule during early spermiogenesis.

SI Tables

Table S1. Prediction of transmembrane helices or a signal peptide in 1700029I15Rik by multiple software. TMD, transmembrane domain; SP, signal peptide.

Software	Prediction of protein type	Reference
TMHMM	Transmembrane protein (TMD: aa 10 - 29)	(14)
SMART	Transmembrane protein (TMD: aa 10 - 29)	(15)
SOSUI	Transmembrane protein (TMD: aa 11 - 33)	(16)
TMPred	Transmembrane protein (TMD: aa 13 - 29)	(17)
HMMTOP	Transmembrane protein (TMD: aa 4 - 25)	(18)
OCTOPUS	Transmembrane protein (TMD: aa 11 - 30)	(19)
Philius	Transmembrane protein (TMD: aa 8 - 29)	(20)
SCAMPI	Transmembrane protein (TMD: aa 9 - 29)	(21)
UniProt	Secreted protein (SP: aa 1 - 29)	(22)
TOPCONS	Secreted protein (SP: aa 1 - 30)	(23)
PolyPhobius	Secreted protein (SP: aa 1 - 31)	(24)
SPOCTOPUS	Secreted protein (SP: aa 1 - 30)	(25)
TMSEG	Secreted protein (SP: aa 1 - 27)	(26)

Table S2. Information about the antibodies used in this study.

Antibodies	Source	Identifier (reference)
Rabbit polyclonal anti-1700029I15Rik antibody	This study	N/A
Mouse monoclonal anti-1D4 antibody	A gift from Robert Molday	N/A (27)
Mouse monoclonal anti-β-actin antibody	Abcam	ab6276
Rabbit polyclonal anti-β-actin antibody	MBL International	PM053
Rat monoclonal anti-ADAM1B	Laboratory of Masahito Ikawa	KS107-158
Mouse monoclonal anti-ADAM3 antibody	Santa Cruz Biotechnology	sc-365288
Mouse monoclonal anti-BASIGIN antibody	Santa Cruz Biotechnology	sc-46700
Rabbit monoclonal anti-CANX antibody	Laboratory of Masahito Ikawa	99051
Rabbit monoclonal anti-CALR antibody	Laboratory of Masahito Ikawa	C1288
Rabbit monoclonal anti-CALR3 antibody	Laboratory of Masahito Ikawa	B6530
Rat monoclonal anti-CD46 antibody	Laboratory of Masahito Ikawa	KS107-116
Rabbit monoclonal anti-CLGN antibody	Laboratory of Masahito Ikawa	83170
Rabbit monoclonal anti-COX4 antibody	Abcam	ab202554
Mouse monoclonal anti-EQTN antibody	A gift from Kiyotaka Toshimori	N/A (28)
Mouse monoclonal anti-FLAG antibody	Sigma-Aldrich	F1804
Rabbit polyclonal anti-FLAG antibody	MBL International	PM020
Rabbit polyclonal anti-GAPDH antibody	Cell Signaling Technology	14C10
Mouse polyclonal anti-GOLGIN97 antibody	Abcam	ab84340
Rabbit polyclonal anti-HSP90B1 antibody	Cell Signaling Technology	2104
Rabbit polyclonal anti-HSPA5 antibody	Affinity BioReagents	PA1-014
Rat monoclonal anti-IZUMO1 antibody	Laboratory of Masahito Ikawa	KS064-125
Mouse monoclonal anti-KDEL antibody	StressGen	SPA-827
Rabbit polyclonal anti-OSTC antibody	ThermoFisher	PA5-32958
Mouse monoclonal anti-P4HB antibody	Affinity BioReagents	MA3-019
Rat monoclonal anti-PA antibody	FUJIFILM Wako	NZ-1
Mouse monoclonal anti-PDIA3 antibody	Abcam	ab13506
Mouse monoclonal anti-RPN2 antibody	Santa Cruz Biotechnology	sc-166421
Rabbit polyclonal anti-SPACA1 antibody	Laboratory of Masahito Ikawa	B9167
Rat monoclonal anti-SPACA4 antibody	Laboratory of Masahito Ikawa	KS139-281
Rabbit polyclonal anti-SPACA6 antibody	This study	N/A
Rabbit polyclonal anti-SYCP1 antibody	Abcam	ab15090
Chicken polyclonal anti-SYCP3 antibody	Laboratory of Hiroki Shibuya	N/A
Rabbit polyclonal anti-TMED10 antibody	Proteintech	15199-1-AP
Rabbit monoclonal anti-TOMM20 antibody	Abcam	ab186735
Mouse monoclonal anti-Ubiquitin antibody	MBL International	MK-11-3
Rabbit polyclonal anti-VDAC3 antibody	Proteintech	14451-1-AP
Mouse monoclonal anti-ZP3R antibody	Invitrogen	MA1-10866
Goat polyclonal anti-ZPBP1 antibody	A gift from Martin M. Matzuk	G176 (29)

Table S3. sgRNAs and primers used in this study.

**Single guide RNA sequences**	
**sgRNA #1**	GGGCGCGAAGGACAGCGTCG; **PAM:** GGG
**sgRNA #2**	CATACTAAATTGCTCTATAC; **PAM:** AGG
**sgRNA #1'**	GAACTGGCACCAGGTTCTGT; **PAM:** CGG
**sgRNA #2'**	GGCTGACCATTATCCTGGGA; **PAM:** AGG
**Primer sequences for genotyping**	
**Fw**	ggcaccaggttctgtcgggc
**Rv**	gtccccaccttgaccggagc
**Fw'**	ggacatgtgtgatagtgctg
**Rv'**	aaggccattgccacttctttg
**Tg_Fw**	ttgagcgggccgcttgcgcactgg
**Tg_Rv**	gccacaccagccaccaccttctg
**Sequences of mutant alleles**[#]	
**Knockout allele** (–619 bp)	GGTTCTCGCCATGCTaaagctattctgggc
**Mutant allele** (–215 bp)	agaactggcaccaggaagggttagttcatg
**Primer sequences for RT-PCR**	
**1700029I15Rik_Fw**	atgcttggggctctgtacc
**1700029I15Rik_Rv**	cggaacagattgctgagacct
**Actb_Fw**	gatgacgatatcgctgcgctg
**Actb_Rv**	gtacgaccagaggcatacagg

[#] Red, sequence upstream of the deletion; purple, sequence downstream of the deletion; UPPERCASE, exon; lowercase, intron or untranslated region

SI Figures

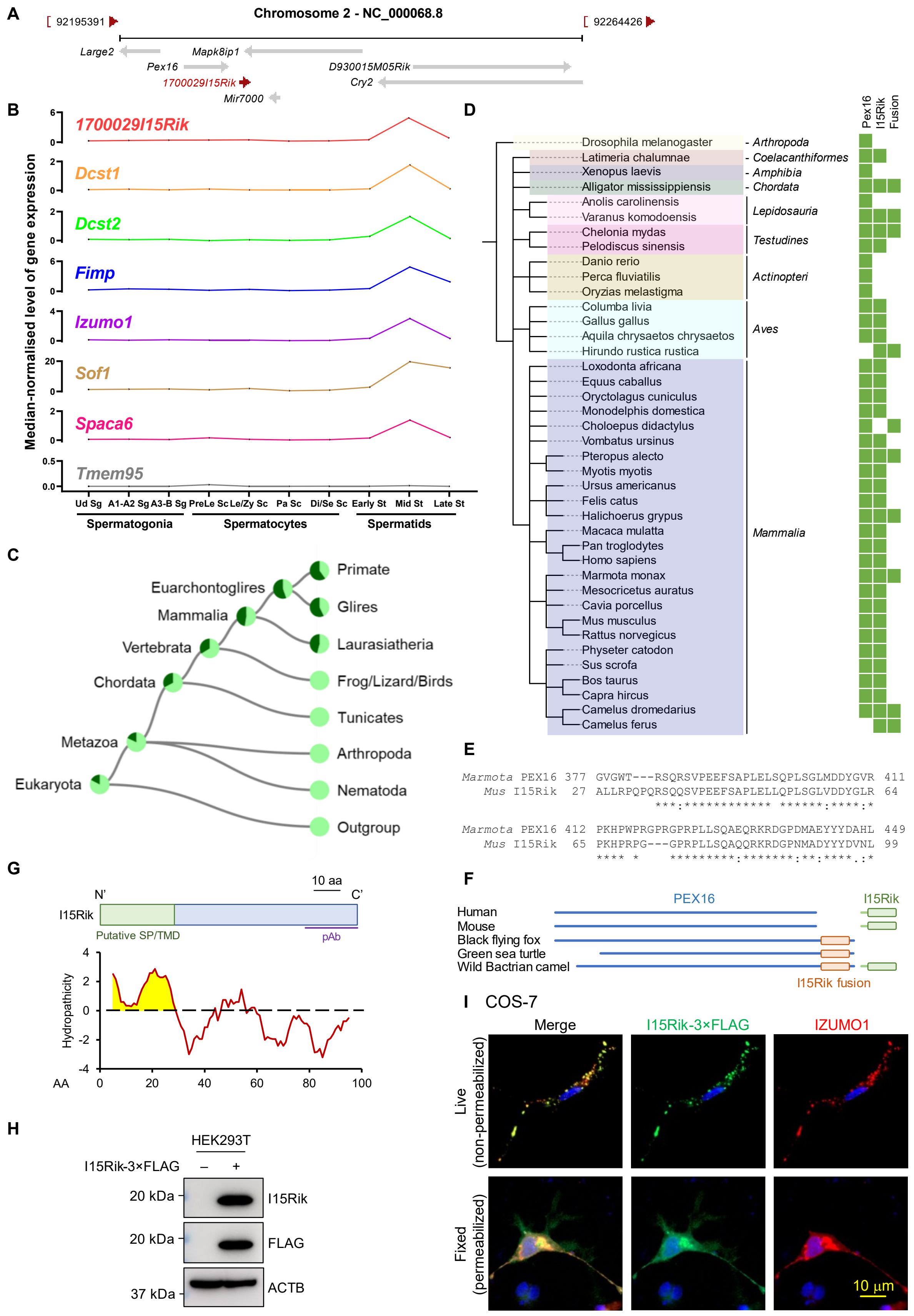
A
Chromosome 2 - NC_000068.8
92195391
92264426
Large2
Mapk8ip1
Pex16
D930015M05Rik
1700029I15Rik
Cry2
Mir7000
B
Median-normalised level of gene expression
1700029I15Rik
Dcst1
Dcst2
Fimp
Izumo1
Sof1
Spaca6
Tmem95
Ud Sg
A1-A2 Sg
A3-B Sg
PreLe Sc
Le/Zy Sc
Pa Sc
Di/Se Sc
Early St
Mid St
Late St
Spermatogonia
Spermatocytes
Spermatids
C
Euarchontoglires
Mammalia
Vertebrata
Chordata
Metazoa
Eukaryota
Primate
Glires
Laurasiatheria
Frog/Lizard/Birds
Tunicates
Arthropoda
Nematoda
Outgroup
D
Pex16
I15Rik
Fusion
Drosophila melanogaster
Latimeria chalumnae
Xenopus laevis
Alligator mississippiensis
Anolis carolinensis
Varanus komodoensis
Chelonia mydas
Pelodiscus sinensis
Danio rerio
Perca fluviatilis
Oryzias melastigma
Columba livia
Gallus gallus
Aquila chrysaetos chrysaetos
Hirundo rustica rustica
Loxodonta africana
Equus caballus
Oryctolagus cuniculus
Monodelphis domestica
Choloepus didactylus
Vombatus ursinus
Pteropus alecto
Myotis myotis
Ursus americanus
Felis catus
Halichoerus grypus
Macaca mulatta
Pan troglodytes
Homo sapiens
Marmota monax
Mesocricetus auratus
Cavia porcellus
Mus musculus
Rattus norvegicus
Physeter catodon
Sus scrofa
Bos taurus
Capra hircus
Camelus dromedarius
Camelus ferus
- Arthropoda
- Coelacanthiformes
- Amphibia
- Chordata
Lepidosauria
Testudines
Actinopteri
Aves
Mammalia
E
Marmota PEX16 377 GVGWT---RSQRSVPEEFSAPLELSQPLSGLMDDYGVR 411
Mus I15Rik 27 ALLRPQPQRSQQSVPEEFSAPLELLQPLSGLVDDYGLR 64
Marmota PEX16 412 PKHPWPRGPRGPRPLLSQAEQRKRDGPDMAEYYYDAHL 449
Mus I15Rik 65 PKHPRPG---GPRPLLSQAQQRKRDGPNMADYYYDVNL 99
F
PEX16
I15Rik
Human
Mouse
Black flying fox
Green sea turtle
Wild Bactrian camel
I15Rik fusion
G
10 aa
N'
C'
I15Rik
Putative SP/TMD
pAb
Hydropathicity
AA
H
HEK293T
I15Rik-3×FLAG
20 kDa
20 kDa
37 kDa
I15Rik
FLAG
ACTB
I
COS-7
Merge
I15Rik-3×FLAG
IZUMO1
Live (non-permeabilized)
Fixed (permeabilized)
10 μm

Fig. S1. Characterization of mouse *1700029I15Rik*. (*A*) Chromosomal localization of mouse *1700029I15Rik*. Adapted from the NCBI database (ncbi.nlm.nih.gov/gene/75641). **(*B*)** Expression of *1700029I15Rik*, *Dcst1*, *Dcst2*, *Fimp*, *Izumo1*, *Sof1*, *Spaca6*, and *Tmem95* in mouse spermatogenic cells based on a scRNA-seq analysis published previously (6). Ud Sg, undifferentiated spermatogonia; A1-A2 Sg, types A1 to A2 spermatogonia; A3-B Sg, types A3 to B spermatogonia; PreLe Sc, preleptotene spermatocytes; Le/Zy Sc, leptotene/zygotene spermatocytes; Pa Sc, pachytene spermatocytes; Di/Se Sc, diplotene/secondary spermatocytes; St, spermatids; Early St, early round spermatids; Mid St, mid-round spermatids; Late St, late round spermatids. **(*C*)** Phylogenetic tree of *1700029I15Rik* generated by TreeFam (30, 31). Dark and light green indicates the presence and loss of orthologs in several species within a taxon, respectively. ***(D)*** Taxonomic tree depicting the potential presence and absence of 1700029I15Rik, PEX16, and PEX16–1700029I15Rik fusion proteins in various vertebrate species. The tree was visualized by iTOL (1). **(*E*)** Sequence alignment of the C-terminal regions of groundhog (*Marmota monax*) PEX16 and mouse (*Mus musculus*) 1700029I15Rik. **(*F*)** Predicted structures of PEX16, 1700029I15Rik, and PEX16–1700029I15Rik fusion proteins in representative vertebrate species. **(*G*)** A predicted structure and hydropathy analysis of mouse 1700029I15Rik. The N terminus of 1700029I15Rik is predicted as either a signal peptide (SP) or a transmembrane domain (TMD) by various algorithms (related to ***SI Appendix*, Table S1**). A polyclonal antibody (pAb) was produced against the C terminus of 1700029I15Rik. Hydropathicity of each amino acid was determined by the Kyte-Doolittle scale (32). The hydrophobic region highlighted in yellow corresponds to the putative SP/TMD. **(*H*)** Western blot detection of 1700029I15Rik-3 × FLAG overexpressed in HEK293T cells. ACTB was analyzed in parallel as a loading control. **(*I*)** *In vitro* topological analysis of 1700029I15Rik by live cell immunostaining. COS-7 cells were transiently transfected with 1700029I15Rik-3 × FLAG and IZUMO1. Live or fixed COS-7 cells were probed with antibodies against FLAG and the ectodomain of IZUMO1, followed by staining with fluorophore-conjugated secondary antibodies. Cell nuclei were visualized by Hoechst 33342.

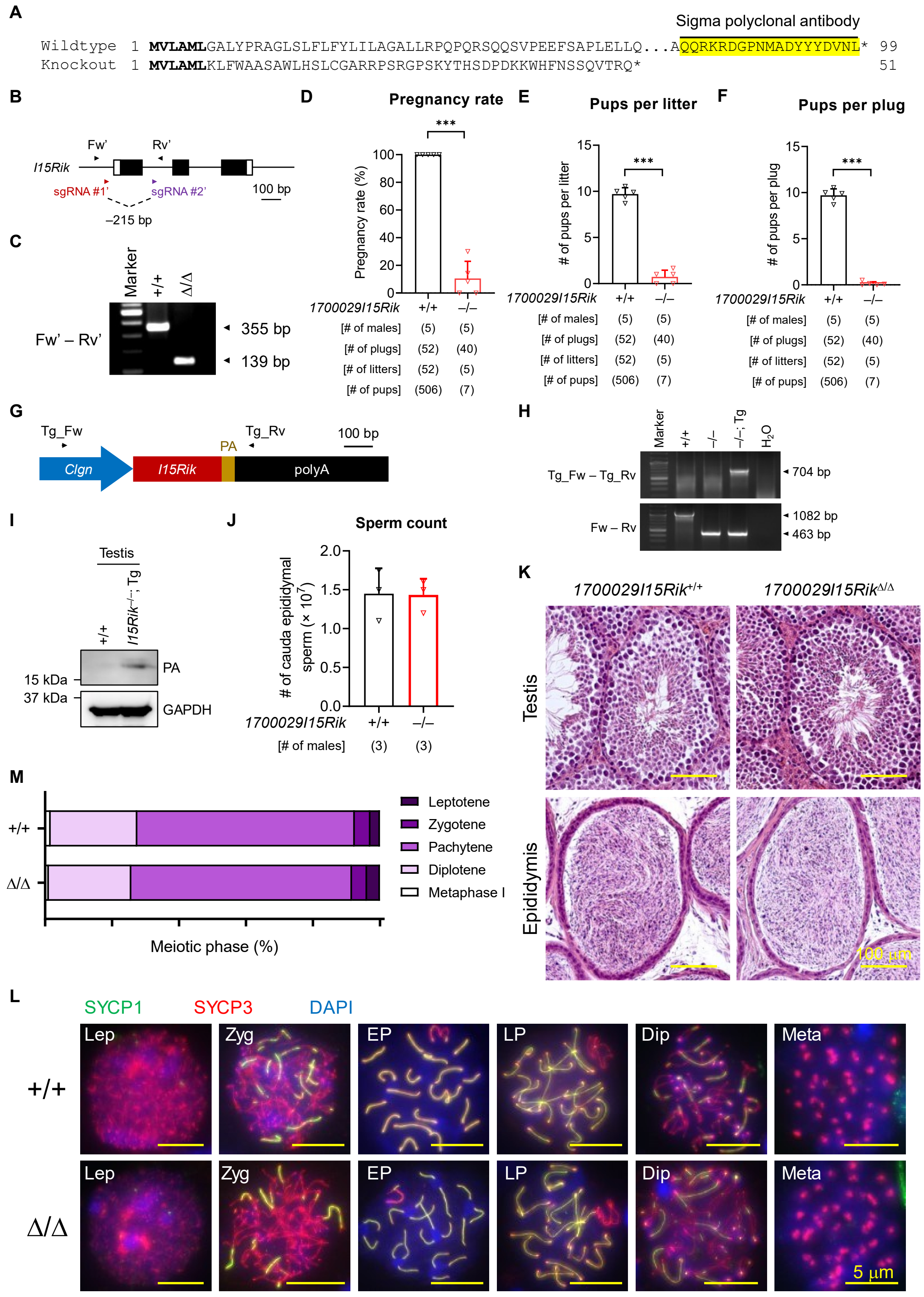

Fig. S2. *1700029I15Rik* mutant male mice are infertile but exhibit normal spermatogenesis. (*A*) Amino acid sequences translated from wildtype and knockout

(*1700029I15Rik*$^{+/-}$) alleles. Identical sequences are highlighted in bold. ***(B)*** Generation of *1700029I15Rik* mutant mice on an inbred genetic background of C57BL/6J by CRISPR/Cas9. SgRNAs #1' and #2' were designed to target the introns upstream and downstream of the first coding exon, respectively. Forward (Fw') and reverse (Rv') primers flanking the deletion region were used for genotyping. ***(C)*** Genomic PCR for detecting wildtype and mutant (*1700029I15Rik*$^{+/\Delta}$) alleles. ***(D – F)*** Fertility tests of wildtype and *1700029I15Rik*$^{-/-}$ males. The male fecundity is represented by pregnancy rate (the sum of litters over the sum of copulation plugs), average number of pups per litter, or the average number of pups per plug. ***(G)*** Generation of a transgenic mouse line expressing C-terminal PA-tagged 1700029I15Rik. A linearized plasmid encoding a *Clgn* promoter, the ORF of *1700029I15Rik* (*I15Rik*), a PA tag, and a rabbit beta-globin polyadenylation (polyA) signal was introduced into zygotes by pronuclear microinjection. ***(H)*** Genomic PCR for detecting the transgene using the forward (Tg_Fw) and reverse (Tg_Rv) primers targeting the *Clgn* promoter and the polyA signal, respectively. ***(I)*** Western blot detection of transgenic expression in the testes. GAPDH was analyzed in parallel as a loading control. ***(J)*** Analysis of sperm counts in wildtype and *1700029I15Rik*$^{-/-}$ male mice. Mature spermatozoa were extracted from cauda epididymides and dispersed in PBS. Sperm counts were measured by hemocytometers. ***(K)*** Analyses of testis and epididymis histology in wildtype and *1700029I15Rik*$^{\Delta/\Delta}$ mice. ***(L)*** Immunostaining of chromosome spreads from wildtype and mutant spermatocytes. The transverse filament and axial element of meiotic chromosomes were immunostained by probing with antibodies against synaptonemal complex protein 1 and 3 (SYCP1 and SYCP3), respectively. The chromatin was visualized by DAPI staining. Lep, leptotene (dotty or discontinuous SYCP3); Zyg, zygotene (linear SYCP3 with partial synapsis); EP, early pachytene (linear SYCP3 with complete synapsis); LP, late pachytene (linear SYCP3 with complete synapsis and thickened SYCP3 ends); Dip, diplotene (linear SYCP3 with desynapsis); Meta, metaphase I (SYCP3 accumulations at centromeres). ***(M)*** Quantification of spermatocytes at various meiotic stages in wildtype and *1700029I15Rik*$^{\Delta/\Delta}$ male mice.

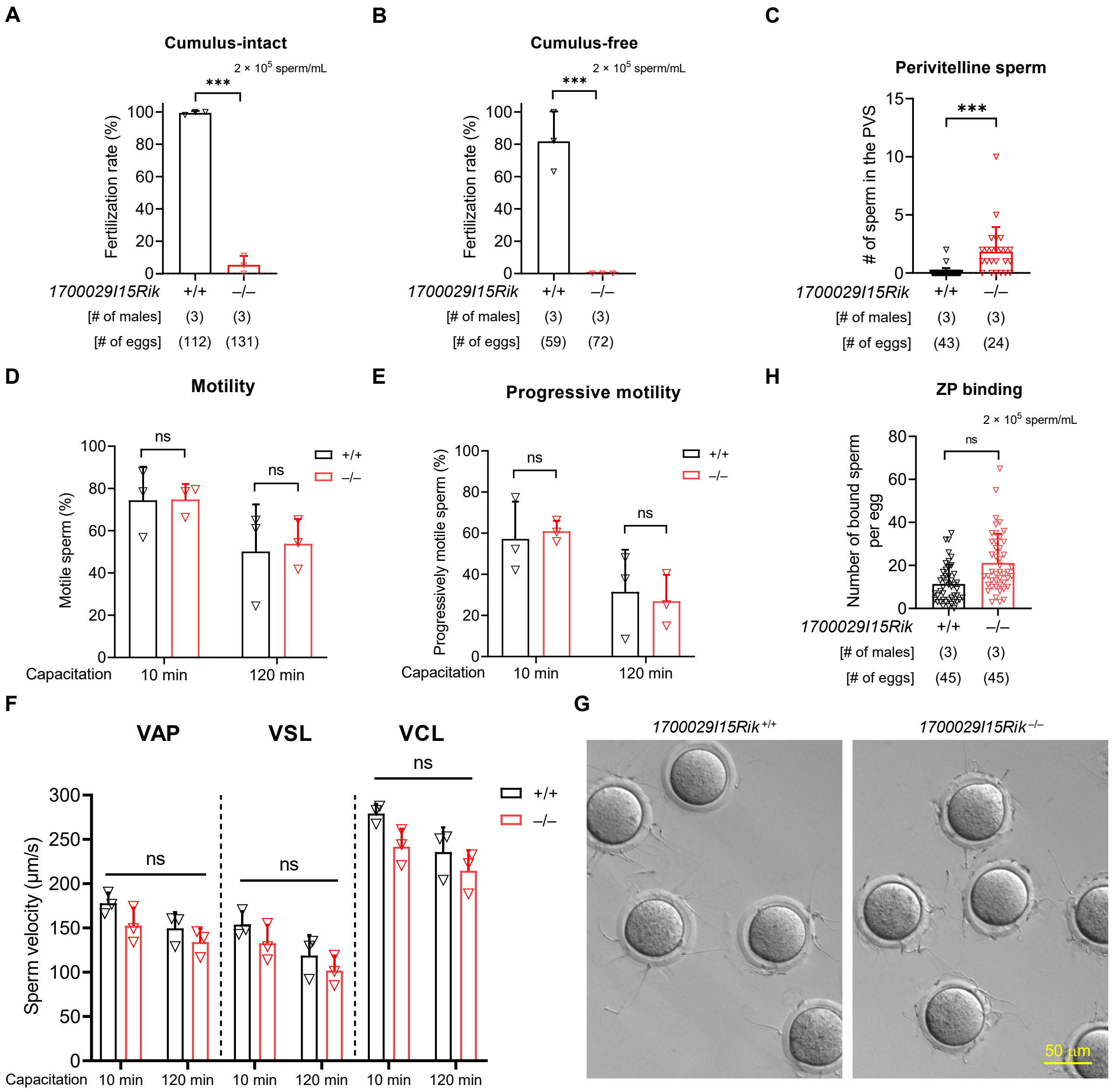

Fig. S3. *1700029I15Rik* knockout sperm show impaired fertilizing ability but normal motility and ZP binding ability. (*A* – *B*) IVF analyses of sperm fertilizing ability using wildtype cumulus-intact and cumulus-free eggs. **(*C*)** Analysis of the numbers of wildtype and knockout sperm accumulated in the egg perivitelline space (related to ***SI Appendix*, Fig. 3*C***). Eggs were harvested from superovulated B6D2F1 female mice that had copulated with wildtype or *1700029I15Rik* knockout males. Perivitelline sperm were visualized by Hoechst 33342 and counted under a fluorescence microscope. **(*D* – *F*)** Analyses of sperm motility, progressive motility, and velocity at 10 and 120 min of incubation in the TYH medium by the Hamilton Thorne CEROS II™ system. VAP, average path velocity; VSL, straight line velocity;

VCL, curvilinear velocity. ns, not significant. **(*G* – *H*)** Analysis of the ZP binding ability of wildtype and knockout spermatozoa.

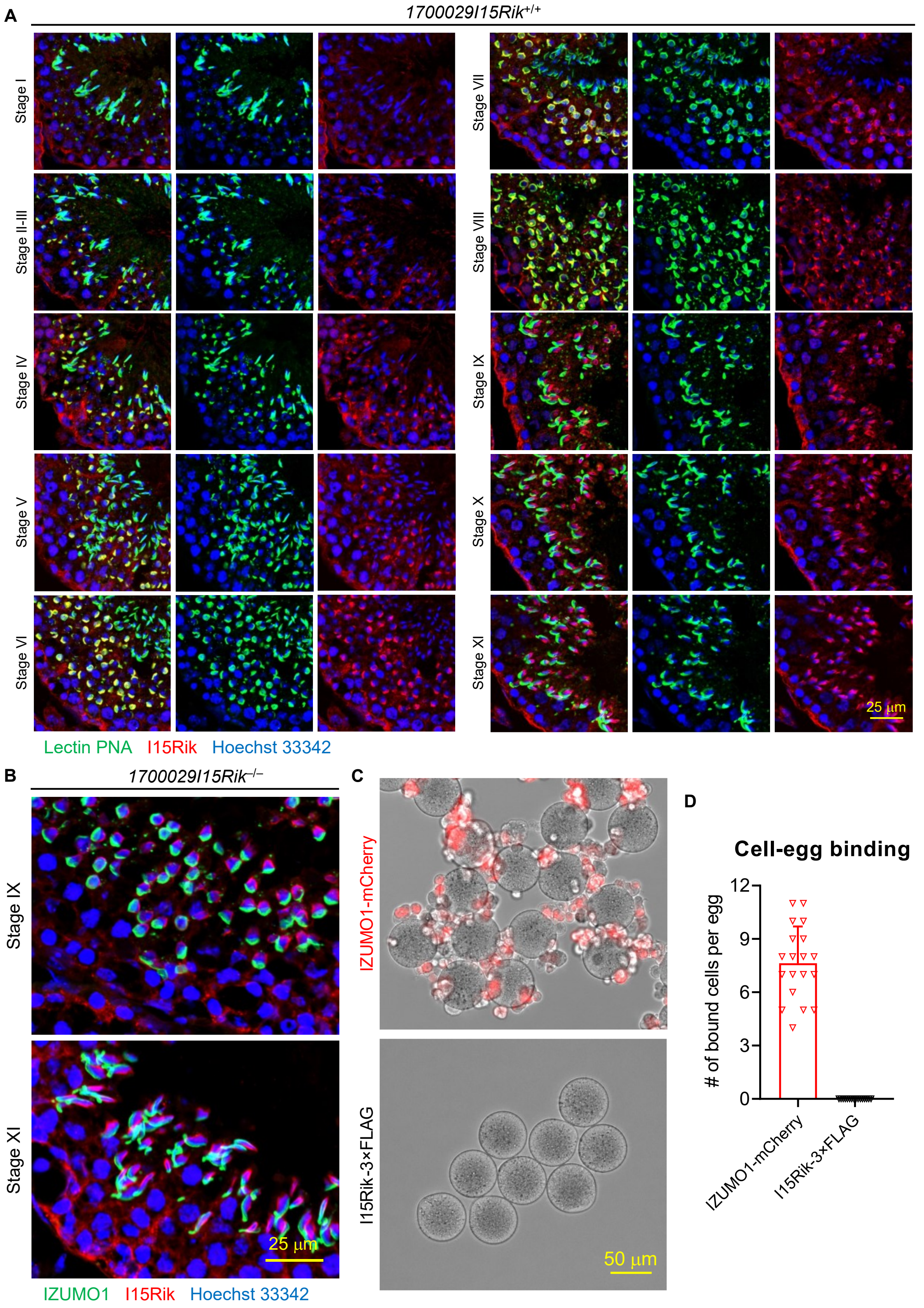

Fig. S4. 1700029I15Rik is localized to the acrosome granule of early round spermatids and absent in elongating spermatids. (*A*) Detailed analysis of the subcellular localization

of 1700029I15Rik in each spermatogenic stage by immunohistochemistry. The acrosome was stained with fluorophore-conjugated lectin peanut agglutinin (PNA) and cell nuclei were visualized by Hoechst 33342. (***B***) Immunostaining of *1700029I15Rik* knockout testis cryosections using the polyclonal antibody against 1700029I15Rik. The antibody non-specifically recognizes the manchette in elongating spermatids. (***C*** – ***D***) Analysis of the oolemma binding ability of HEK293T cells transiently expressing IZUMO1-mCherry or 1700029I15Rik-3 × FLAG.

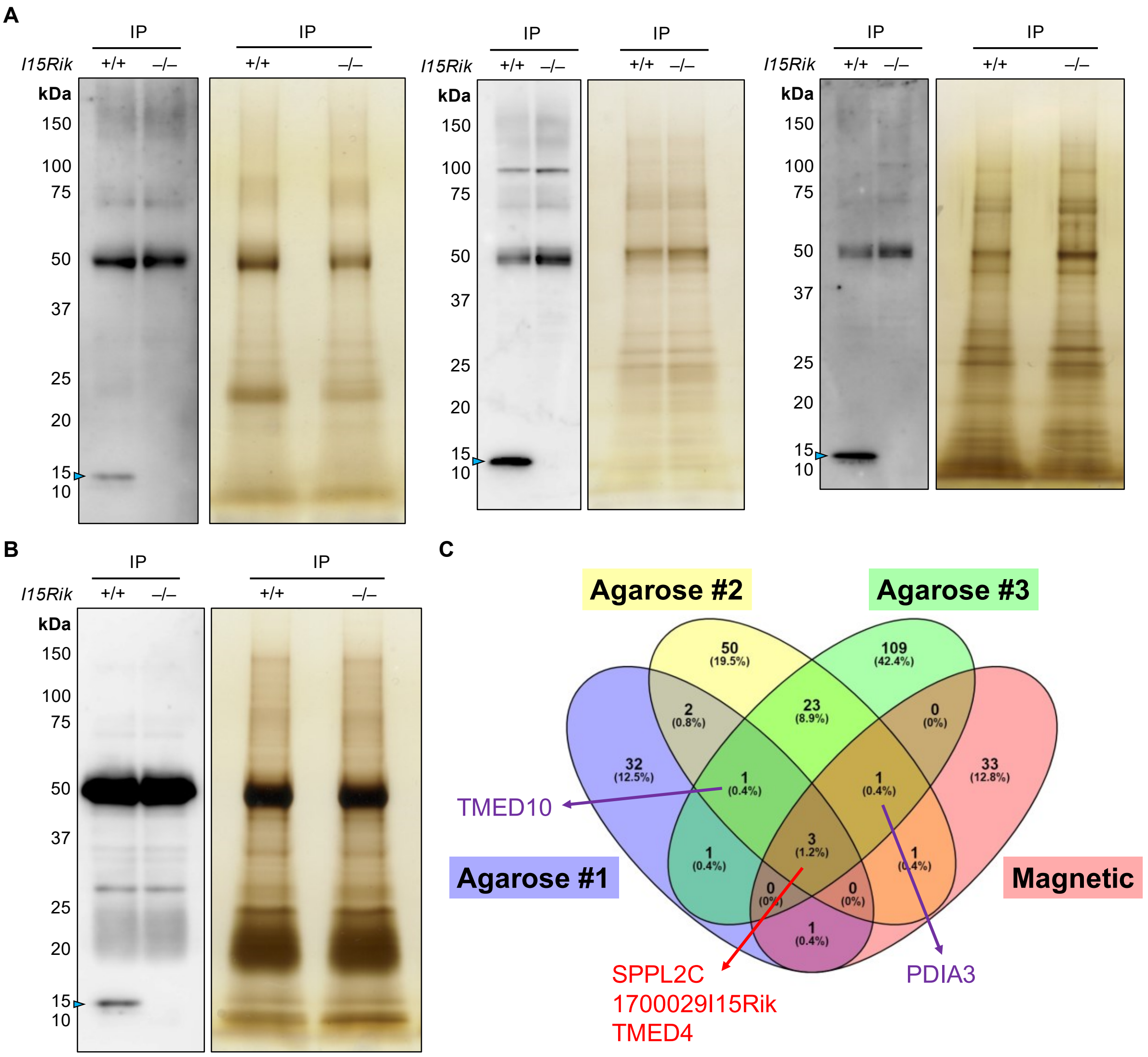

D

Relevance	Protein symbol	Agarose +/+ #1	Agarose −/− #1	Agarose +/+ #2	Agarose −/− #2	Agarose +/+ #3	Agarose −/− #3	Magnetic +/+	Magnetic −/−	Description
4	SPPL2C	22	0	27	0	19	0	6	0	An ER-resident signal peptide peptidase that cleaves type II transmembrane proteins
4	1700029I15Rik	3	0	2	0	2	0	5	0	Self
4	TMED4	1	0	1	0	2	0	1	0	An ERGIC protein involved in ER–Golgi vesicular protein trafficking
3	TMED10	2	0	3	0	1	0	6	5	An ERGIC protein involved in ER–Golgi vesicular protein trafficking and protein quality control in the ER and Golgi apparatus
3	PDIA3	2	2	12	0	10	0	1	0	A protein disulfide isomerase implicated in protein folding and gamete fusion
3	VDAC3	4	1	1	0	3	0	6	3	A mitochondrial outer membrane protein involved in mitochondrial metabolism
2.5	RPN2	12	0	9	0	4	2	19	15	A subunit of the OST complex involved in *N*-linked glycosylation
2.5	OSTC	1	0	2	0	-	-	2	1	A subunit of the OST complex involved in *N*-linked glycosylation
2.5	DAD1	-	-	2	0	2	0	3	1	A subunit of the OST complex involved in *N*-linked glycosylation

Fig. S5. 1700029I15Rik interacts with ER proteins implicated in *N*-linked glycosylation, disulfide isomerization, and ER–Golgi vesicular trafficking. (*A*) Silver stained SDS-PAGE gels of proteins immunoprecipitated from wildtype and knockout testes. Co-IP was

performed using the anti-1700029I15Rik antibody that was covalently crosslinked to the agarose resin. 1700029I15Rik (blue arrowhead) was detected in the wildtype but absent in the knockout eluates by Western blotting. Three biological replicates were performed for the co-IP/MS experiments to validate the reproducibility. **(*B*)** Silver stained SDS-PAGE gels of proteins immunoprecipitated from wildtype and knockout testes using antibody-conjugated magnetic beads. The anti-1700029I15Rik antibody was not covalently crosslinked to the magnetic beads. **(*C*)** Venn diagram depicting proteins commonly identified in the four replicates of the co-IP/MS experiments. Only the proteins specifically detected in the wildtype samples are included. **(*D*)** Proteins tightly associated with 1700029I15Rik as revealed by the co-IP/MS analyses. The relevance of each protein was scored based on the spectral counts detected in wildtype and knockout samples [1 for exclusive detection in the wildtype sample; 0.5 for fold changes (wildtype over knockout) ≥ 2; 0 for fold changes < 2].

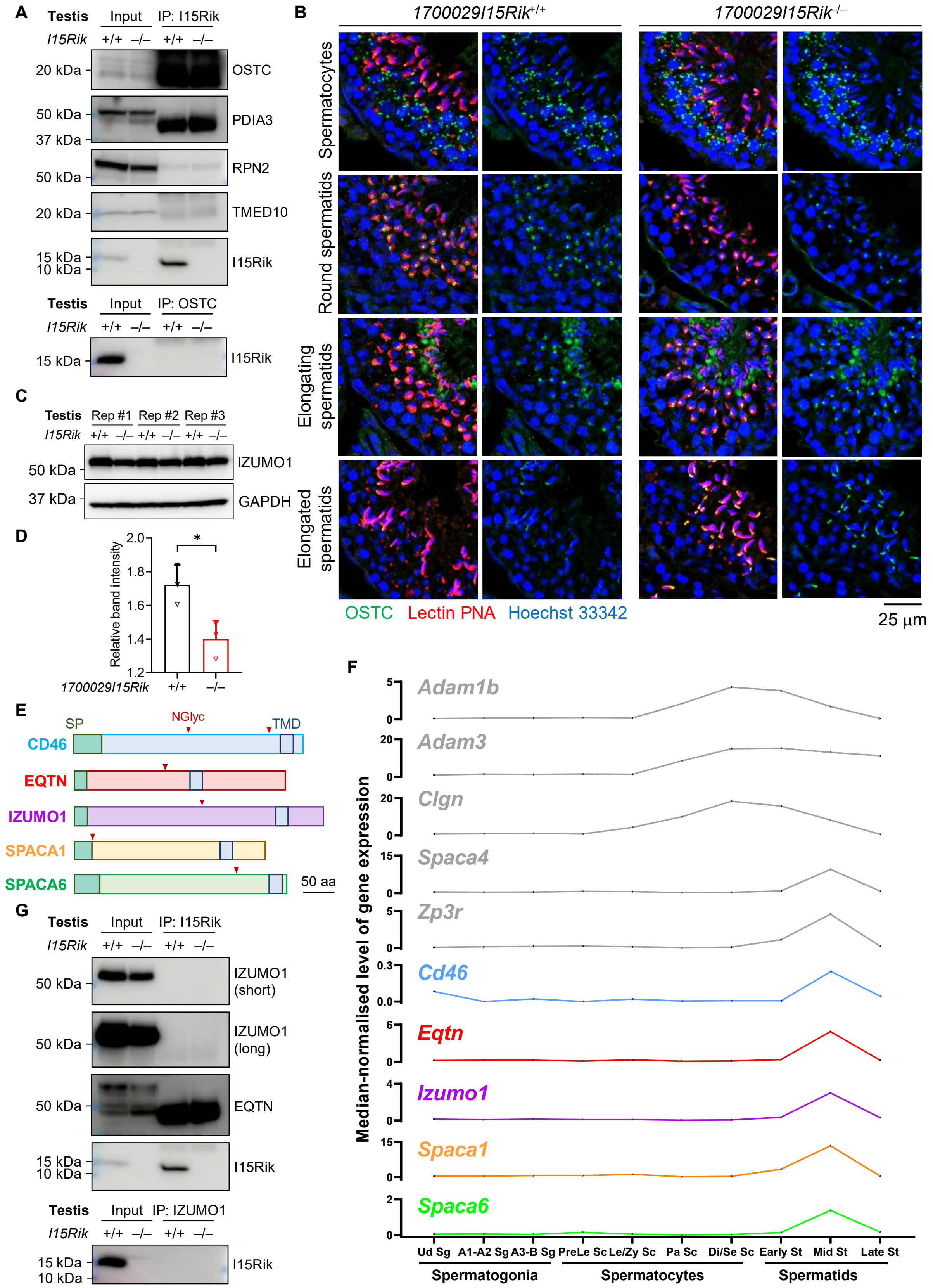

Fig. S6. Ablation of 1700029I15Rik results in downregulation of multiple acrosomal membrane proteins in the testes. (*A*) Analyses of the interactions between 1700029I15Rik

and multiple ER proteins by co-IP coupled with Western blotting. Co-IP was performed on wildtype and knockout testis lysates using antibody-conjugated magnetic beads. **(*B*)** Analysis of subcellular localization of OSTC in wildtype and *1700029I15Rik* knockout testes by immunohistochemistry. The acrosome was stained with fluorophore-conjugated lectin PNA and cell nuclei were visualized by Hoechst 33342. **(*C* – *D*)** Western blot detection of IZUMO1 in wildtype and knockout testes. Three biological replicates (Rep) were performed to demonstrate the reproducibility. GAPDH was analyzed in parallel as a loading control. The protein band intensities were measure by ImageJ and the band intensities of IZUMO1 relative to GAPDH were calculated. **(*E*)** Protein structures of CD46, EQTN, IZUMO1, SPACA1, and SPACA6. The signal peptide (SP; colored in teal), transmembrane domain (TMD; colored in blue), and *N*-linked glycosylation (NGlyc) sites (red arrowheads) are highlighted. **(*F*)** Expression of *Adam1b*, *Adam3*, *Clgn*, *Cd46*, *Eqtn*, *Izumo1*, *Spaca1*, *Spaca4*, and *Spaca6*, and *Zp3r* in the mouse spermatogenic cells based on a published scRNA-seq dataset (6). Ud Sg, undifferentiated spermatogonia; A1-A2 Sg, types A1 to A2 spermatogonia; A3-B Sg, types A3 to B spermatogonia; PreLe Sc, preleptotene spermatocytes; Le/Zy Sc, leptotene/zygotene spermatocytes; Pa Sc, pachytene spermatocytes; Di/Se Sc, diplotene/secondary spermatocytes; St, spermatids; Early St, early round spermatids; Mid St, mid-round spermatids; Late St, late round spermatids. **(*G*)** Analyses of the interactions between 1700029I15Rik and IZUMO1 or EQTN by co-IP coupled with Western blotting. Co-IP was performed on wildtype and knockout testis lysates using antibody-conjugated magnetic beads. These co-IP and Western blot experiments were performed together with the ones shown in ***SI Appendix*, Fig. S6*A*** and share a same control (co-IP with the 1700029I15Rik antibody followed by Western blot detection of 1700029I15Rik).

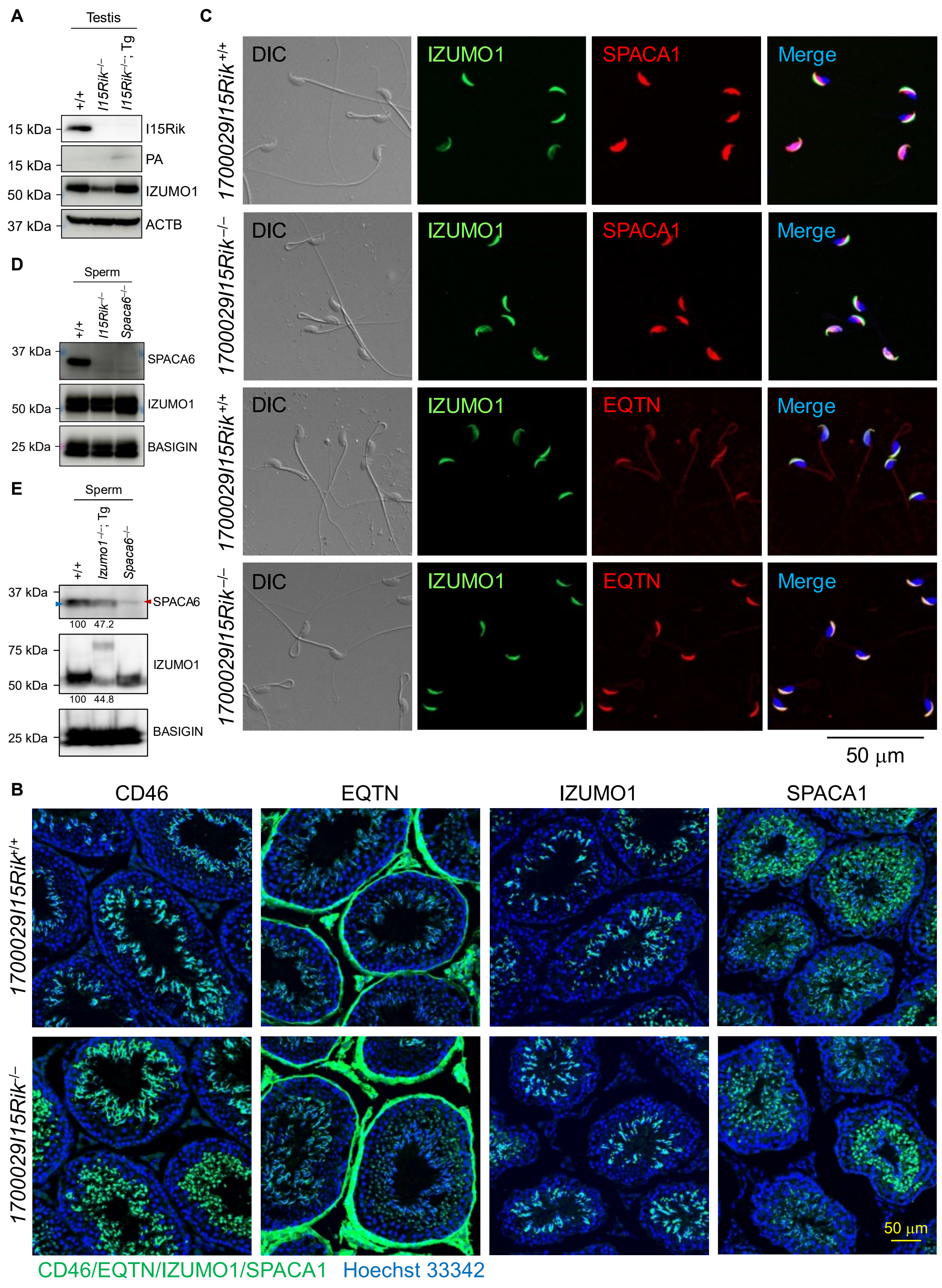

Fig. S7. The downregulated acrosomal membrane proteins show normal localization in *1700029I15Rik* knockout testes and sperm. (*A*) Western blot detection of

1700029I15Rik, 1700029I15Rik-PA, and IZUMO1 in wildtype, *1700029I15Rik*$^{-/-}$, and *1700029I15Rik*$^{-/-}$; Tg testes. ACTB was analyzed as a loading control. (***B***) Immunohistochemistry analyses of CD46, EQTN, IZUMO1, and SPACA1 in wildtype and knockout testes. Cell nuclei were visualized by Hoechst 33342 staining. (***C***) Immunocytochemistry analyses of EQTN, IZUMO1, and SPACA1 in wildtype and knockout spermatozoa. Sperm nuclei were stained with Hoechst 33342. (***D***) Western blot analysis of SPACA6 in wildtype, *1700029I15Rik* knockout, and *Spaca6* knockout spermatozoa. IZUMO1 and BASIGIN were analyzed in parallel. (***E***) Western blot analyses of SPACA6 and IZUMO1 in the sperm of wildtype, *Izumo1*$^{-/-}$; Tg, and *Spaca6*$^{-/-}$ males. Blue and red arrowheads indicate the SPACA6 band and a non-specific band introduced by immunoglobulin G (IgG) contamination, respectively. Protein band intensities were measured by ImageJ. The band intensities relative to BASIGIN were calculated, with the wildtype bands being set as 100%.

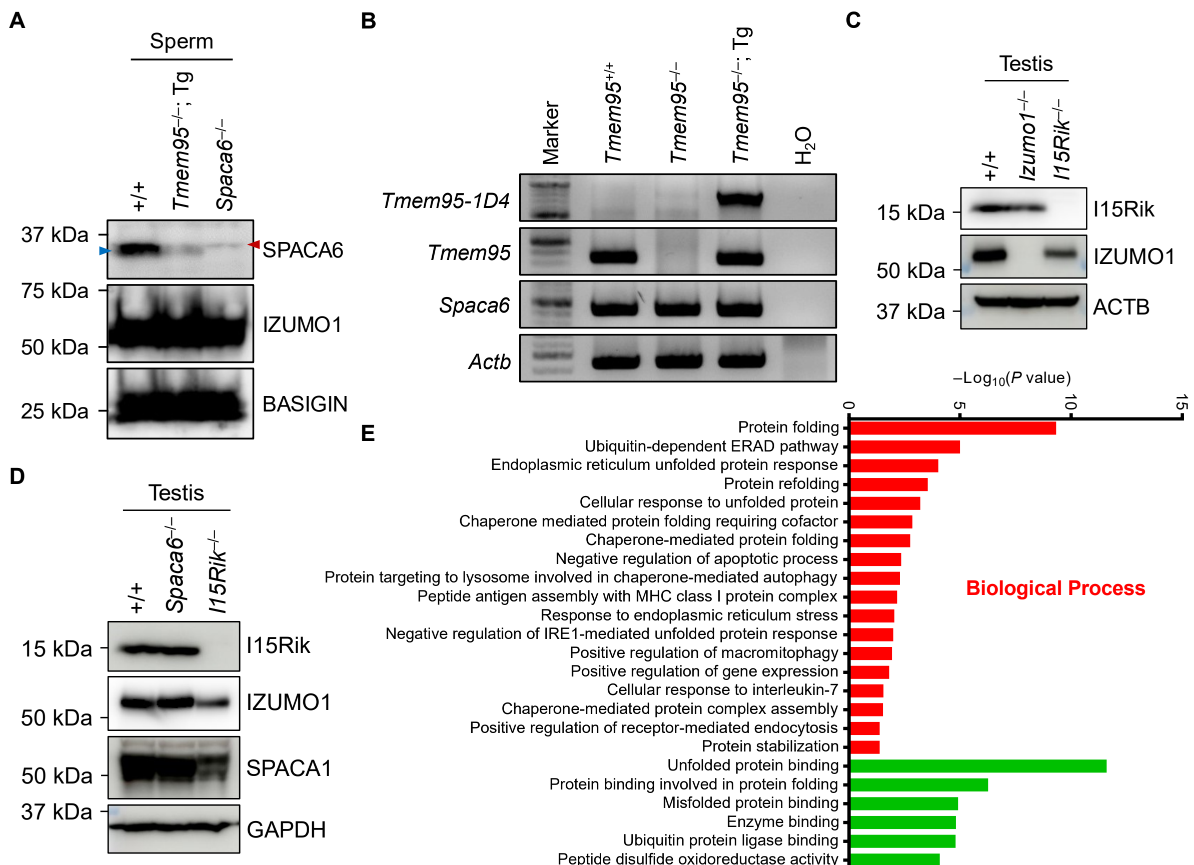

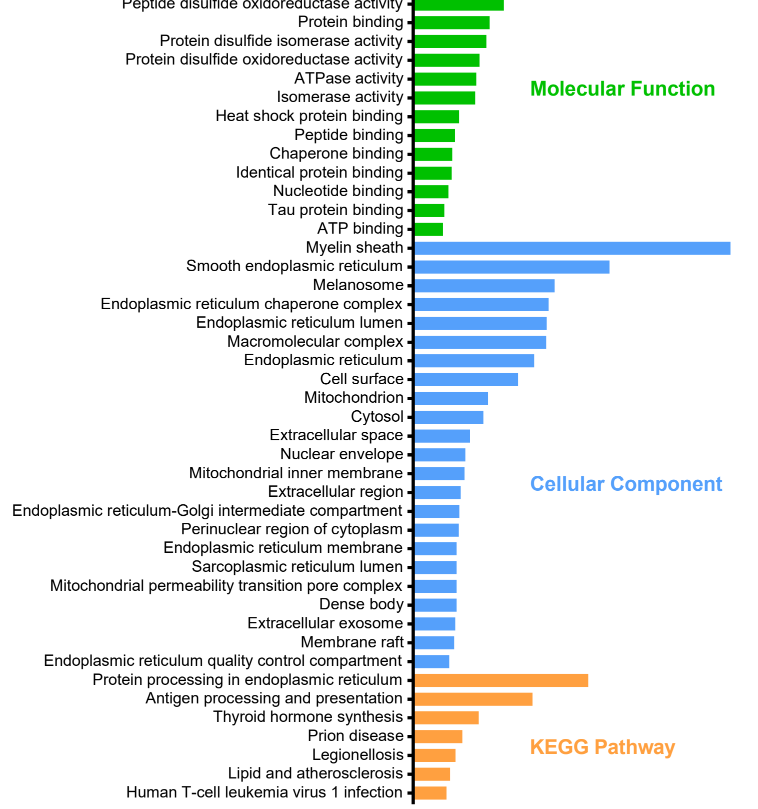

F Downregulated sperm proteins

Protein symbol	Normalized total spectra		Fold change
	Wildtype	Knockout	
PSMA1	13	2	0.153853
RNLS	45	0	2.22e-06
ACR	38	23	0.605264
DPEP3	23	10	0.434785
PRSS39	22	10	0.454548
ACSL1	32	16	0.500002
FAHD2A	28	16	0.57143
OXCT2A	107	82	0.766355
SUCLA2	52	34	0.653847

Acrosomal vesicle (GO:0001669)
Mitochondrion (GO:0005739)

G Acrosomal membrane proteins

Protein symbol	Normalized total spectra		Fold change
	Wildtype	Knockout	
CD46	6	7	1.165426
EQTN	13	7	0.537891
IZUMO1	31	22	0.708925
SPACA1	21	12	0.570846

Fig. S8. Depletion of *1700029I15Rik* leads to upregulation of multiple ER chaperones in the sperm. (***A***) Western blot detection of SPACA6 in the sperm of wildtype, *Tmem95*$^{-/-}$;

Tg, and *Spaca6*$^{-/-}$ male mice. IZUMO1 and BASIGIN were analyzed in parallel. Blue and red arrowheads indicate the SPACA6 band and a non-specific band introduced by IgG contamination, respectively. (***B***) RT-PCR depicting the mRNA expression of the *Tmem95-1D4* transgene, *Tmem95*, and *Spaca6* in wildtype, *Tmem95*$^{-/-}$, *Tmem95*$^{-/-}$; Tg testes. The expression of *Actb* was analyzed in parallel as a loading control. (***C*** – ***D***) Western blot analyses of 1700029I15Rik in *Izumo1*$^{-/-}$ and *Spaca6*$^{-/-}$ testes. ACTB or GAPDH was analyzed as a loading control. (***E***) GO and KEGG analyses of proteins upregulated in the knockout spermatozoa using the DAVID knowledgebase (33). (***F***) Representative proteins downregulated in *1700029I15Rik* knockout spermatozoa. The proteins localized to the acrosomal vesicles (GO:0001669) or mitochondria (GO:0005739) are highlighted in light yellow and light blue, respectively. (***G***) MS analysis depicting the levels of several acrosomal membrane proteins in wildtype and *1700029I15Rik* knockout sperm. EQTN, and IZUMO1, and SPACA1 show less spectral counts in the knockout sperm. However, the differences are not statistically significant as determined by Fisher's exact test in Scaffold 5 software (Proteome Software, Portland, OR).

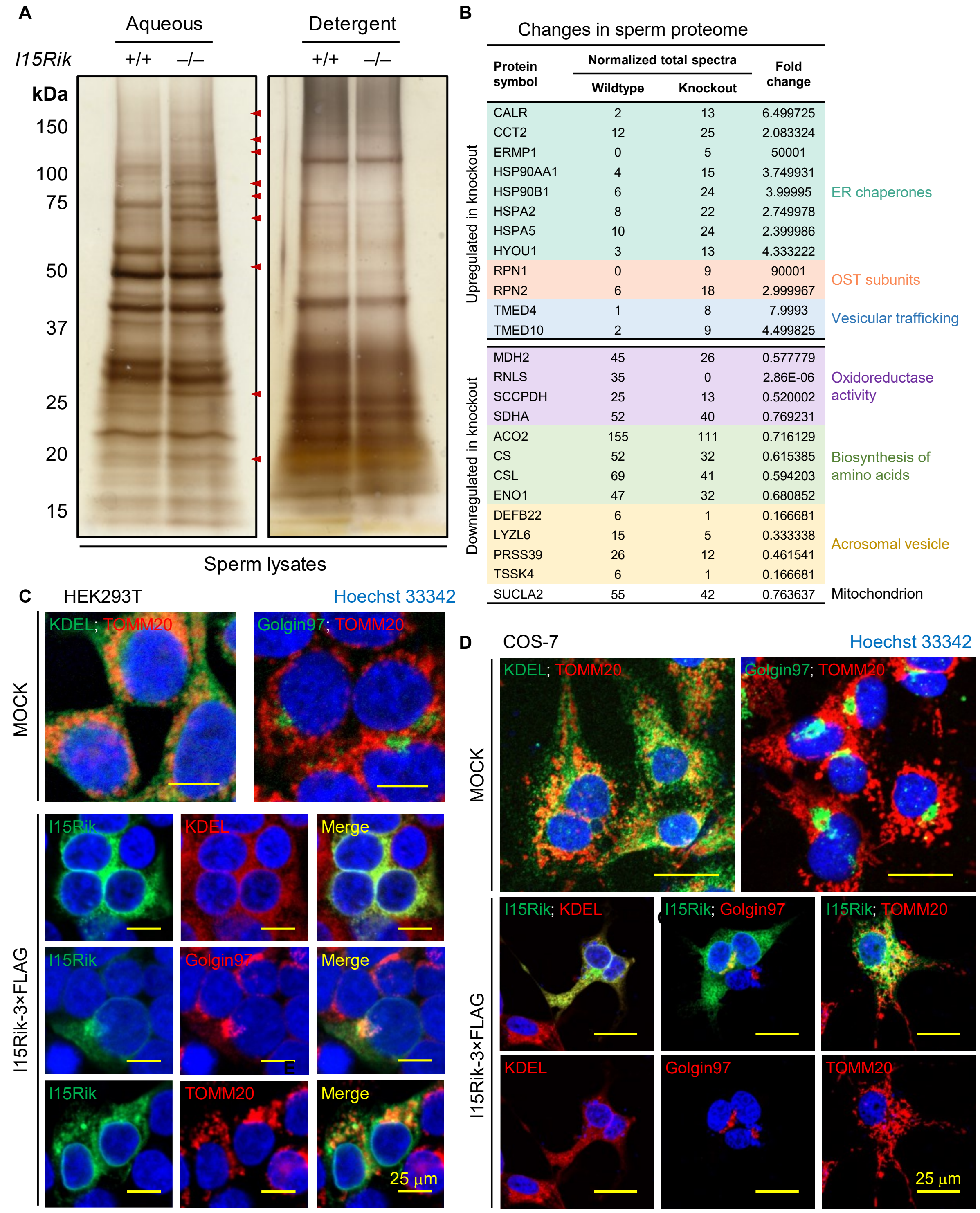

Fig. S9. Loss of *1700029I15Rik* causes upregulation of multiple ER chaperones in the sperm. (*A*) Silver stained SDS-PAGE gels of wildtype and knockout sperm proteins fractionated by Triton X-114. Red arrowheads indicate protein bands specifically detected in the knockout sperm. **(*B*)** Proteomic changes detected by MS analyses on Triton X-114-fractionated wildtype and knockout sperm proteins. Among the proteins upregulated in the

knockout sperm, multiple ERAD-associated ER chaperones (highlighted in teal), the OST subunits RPN1 and RPN2 (colored in light orange), and the ERGIC proteins TMED4 and TMED10 (colored in blue), were detected. **(*C*)** Immunostaining of fixed HEK293T cells transiently expressing 1700029I15Rik-3 × FLAG. The ER, mitochondria, and Golgi apparatus were visualized using antibodies against KDEL (34), translocase of outer mitochondrial membrane 20 (TOMM20) (35), and golgin subfamily A member 1 (Golga1, also known as Golgin97) (36), respectively. The cell nuclei were visualized by Hoechst 33342. **(*D*)** Immunostaining of fixed COS-7 cells transiently expressing 1700029I15Rik-3 × FLAG. The ER, mitochondria, and Golgi apparatus were visualized using antibodies against KDEL, TOMM20, and Golgin97, respectively. Cell nuclei were stained with Hoechst 33342.

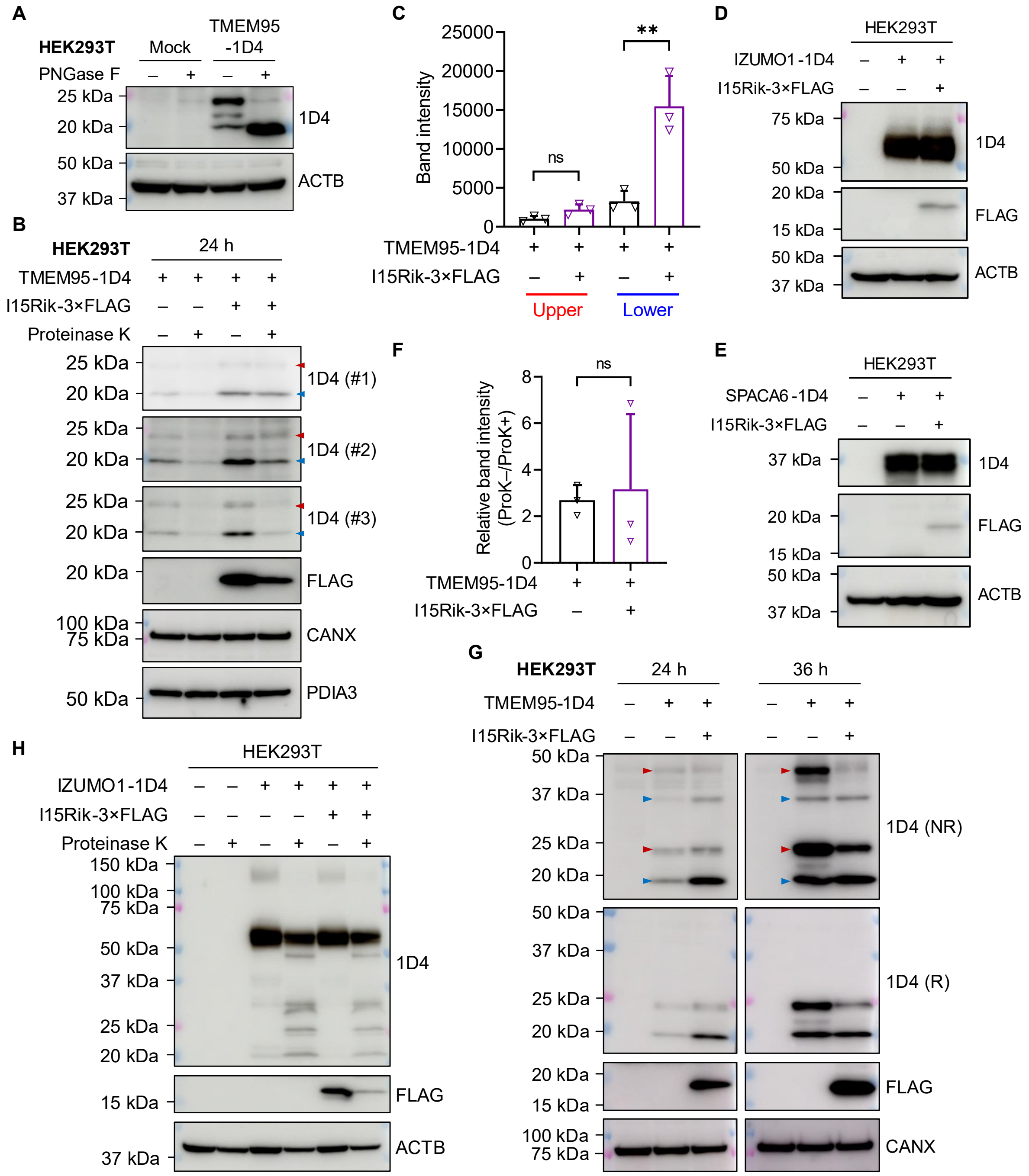

Fig. S10. 1700029I15Rik facilitates the biosynthesis of acrosomal membrane proteins. (***A***) *N*-linked glycan analysis of TMEM95 in HEK293T cells. The protein lysates were treated with Peptide-*N*-Glycosidase F (PNGase F) to cleave the *N*-linked glycan sidechains. ACTB was analyzed as a loading control. TMEM95 has two *N*-linked glycosylation sites. From top to bottom, the three protein bands detected in the untreated sample represent fully glycosylated, partially glycosylated, and non-glycosylated TMEM95. (***B***) *In vitro* analysis of the expression and localization of TMEM95 with or without the presence of 1700029I15Rik.

HEK293T cells transiently expressing TMEM95-1D4 and 1700029I15Rik-3 × FLAG were treated with proteinase K at 24 h post transfection and harvested for Western blot analyses. CANX and PDIA3 were analyzed in parallel as loading controls. Red and blue arrowheads indicate fully glycosylated and non-glycosylated TMEM95, respectively. **(*C*)** Quantitative analysis of the expression levels of fully glycosylated (Upper) and non-glycosylated (Lower) TMEM95 with or without the presence of 1700029I15Rik (related to ***SI Appendix*, Fig. S10*B***). The protein band intensities were measured by ImageJ. **(*D* – *E*)** *In vitro* analyses of the expression of IZUMO1 and SPACA6 in the presence of 1700029I15Rik. HEK293T cells were harvested at 24 h post transfection for Western blot analyses. **(*F*)** Quantitative analysis of the surface expression of TMEM95 with or without the presence of 1700029I15Rik (related to ***SI Appendix*, Fig. S10*B***). Relative band intensities were calculated by dividing the intensity of the untreated group with that of the proteinase K (ProK)-treated group. **(*G*)** *In vitro* analysis of TMEM95 co-expressed with 1700029I15Rik. HEK293T cells transiently expressing TMEM95-1D4 and 1700029I15Rik-3 × FLAG were harvested at 24 h and 36 h post transfection for Western blot analyses. TMEM95 was immunodetected using an anti-1D4 antibody under non-reducing and non-denaturing (NR) and reducing and denaturing (R) conditions. CANX was analyzed as a loading control. Red and blue arrowheads indicate fully glycosylated and non-glycosylated TMEM95, respectively. **(*H*)** *In vitro* analyses of the surface expression of IZUMO1 in the presence of 1700029I15Rik. HEK293T cells transiently expressing IZUMO1 and 1700029I15Rik-3 × FLAG were treated with proteinase K at 24 h post transfection and then harvested for Western blot analyses.

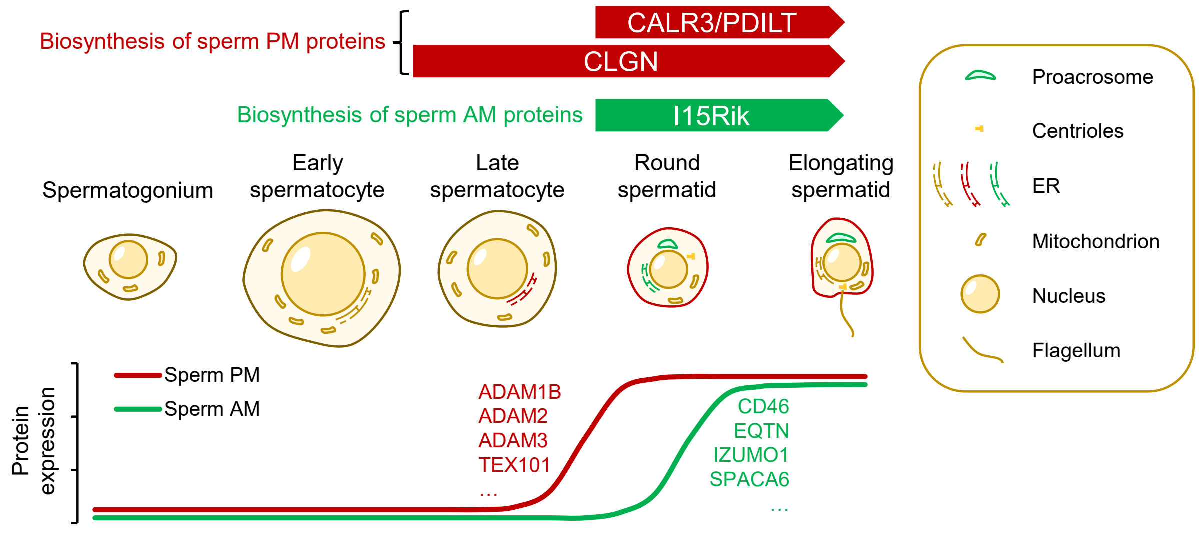

Fig. S11. A diagram depicting the spatiotemporal regulation of the biosynthesis of sperm plasma membrane and acrosomal membrane proteins. Sperm plasma membrane (PM) proteins, such as ADAM1B, ADAM2, and ADAM3, show peak expression in spermatocytes, whereas acrosomal membrane (AM) protein, such as CD46, EQTN, and IZUMO1, are predominantly expressed in mid-round spermatids. Corresponding to their putative substrates, CLGN and 1700029I15Rik exhibit biased expression in late spermatocytes (highlighted in red in the ER) and early spermatids (highlighted in green in the ER), respectively. CALR3 and PDILT work with CLGN in the processing of sperm plasma membrane proteins and are expressed during spermiogenesis (37, 38). The two protein biosynthesis pathways act independently during spermatogenesis, because *Clgn* knockout sperm show ability to fuse with wildtype eggs and *1700029I15Rik* knockout sperm can pass through the uterotubal junction and bind to the ZP. The upper arrow banners represent the expression timing of 1700029I15Rik, CALR3, CLGN, and PDILT proteins based on previous Western blot analyses or the published scRNA-seq data (6, 37). The lower curve graph indicates the estimated protein levels during spermatogenesis based on the patterns of mRNA expression. In the round and elongating spermatids, the plasma and acrosomal membranes are colored in red and green, respectively, to represent the expression timing of the membrane proteins.

SI Legend for Dataset S1

Dataset S1. Mass spectrometric analyses in this study. Data were processed by Scaffold 5 software (Proteome Software, Portland, OR).

SI References

1. Letunic I & Bork P (2021) Interactive Tree Of Life (iTOL) v5: an online tool for phylogenetic tree display and annotation. *Nucleic Acids Res.* 49(W1):W293-W296.
2. Schoch CL*, et al.* (2020) NCBI Taxonomy: a comprehensive update on curation, resources and tools. *Database* 2020:baaa062.
3. Rozewicki J, Li S, Amada KM, Standley DM, & Katoh K (2019) MAFFT-DASH: integrated protein sequence and structural alignment. *Nucleic Acids Res.* 47(W1):W5-W10.
4. Jumper J*, et al.* (2021) Highly accurate protein structure prediction with AlphaFold. *Nature* 596(7873):583-589.
5. Ashkenazy H*, et al.* (2016) ConSurf 2016: an improved methodology to estimate and visualize evolutionary conservation in macromolecules. *Nucleic Acids Res.* 44(W1):W344-W350.
6. Hermann BP*, et al.* (2018) The Mammalian Spermatogenesis Single-Cell Transcriptome, from Spermatogonial Stem Cells to Spermatids. *Cell Rep.* 25(6):1650-1667.
7. Taguchi Y & Schätzl HM (2014) Small-scale Triton X-114 Extraction of Hydrophobic Proteins. *Bio-protocol* 4(11):e1139.
8. Lu Y*, et al.* (2019) CRISPR/Cas9-mediated genome editing reveals 30 testis-enriched genes dispensable for male fertility in mice. *Biol. Reprod.* 101(2):501-511.
9. Noda T*, et al.* (2020) Sperm proteins SOF1, TMEM95, and SPACA6 are required for sperm–oocyte fusion in mice. *Proc. Natl. Acad. Sci. U. S. A.* 117(21):11493-11502.

10. Perez-Riverol Y*, et al.* (2018) The PRIDE database and related tools and resources in 2019: improving support for quantification data. *Nucleic Acids Res.* 47(D1):D442-D450.

11. Tang S*, et al.* (2022) Human sperm TMEM95 binds eggs and facilitates membrane fusion. *Proc. Natl. Acad. Sci. U. S. A.* 119(40):e2207805119.

12. Moreno RD, Ramalho-Santos Jo, Sutovsky P, Chan EKL, & Schatten G (2000) Vesicular Traffic and Golgi Apparatus Dynamics During Mammalian Spermatogenesis: Implications for Acrosome Architecture. *Biol. Reprod.* 63(1):89-98.

13. Au CE*, et al.* (2015) Expression, sorting, and segregation of Golgi proteins during germ cell differentiation in the testis. *Mol. Biol. Cell* 26(22):4015-4032.

14. Krogh A, Larsson B, von Heijne G, & Sonnhammer ELL (2001) Predicting transmembrane protein topology with a hidden markov model: application to complete genomes. *J. Mol. Biol.* 305(3):567-580.

15. Letunic I, Khedkar S, & Bork P (2020) SMART: recent updates, new developments and status in 2020. *Nucleic Acids Res.* 49(D1):D458-D460.

16. Mitaku S, Hirokawa T, & Tsuji T (2002) Amphiphilicity index of polar amino acids as an aid in the characterization of amino acid preference at membrane–water interfaces. *Bioinformatics* 18(4):608-616.

17. Hofmann K & Stoffel W (1993) TMbase-A database of membrane spanning proteins segments. *Biol. Chem. Hoppe-Seyler* 374:166.

18. Tusnády GE & Simon I (2001) The HMMTOP transmembrane topology prediction server. *Bioinformatics* 17(9):849-850.

19. Viklund H & Elofsson A (2008) OCTOPUS: improving topology prediction by two-track ANN-based preference scores and an extended topological grammar. *Bioinformatics* 24(15):1662-1668.

20. Reynolds SM, Käll L, Riffle ME, Bilmes JA, & Noble WS (2008) Transmembrane Topology and Signal Peptide Prediction Using Dynamic Bayesian Networks. *PLoS Comp. Biol.* 4(11):e1000213.

21. Bernsel A*, et al.* (2008) Prediction of membrane-protein topology from first principles. *Proc. Natl. Acad. Sci. U. S. A.* 105(20):7177-7181.

22. Consortium TU (2020) UniProt: the universal protein knowledgebase in 2021. *Nucleic Acids Res.* 49(D1):D480-D489.

23. Tsirigos KD, Peters C, Shu N, Käll L, & Elofsson A (2015) The TOPCONS web server for consensus prediction of membrane protein topology and signal peptides. *Nucleic Acids Res.* 43(W1):W401-W407.

24. Käll L, Krogh A, & Sonnhammer ELL (2005) An HMM posterior decoder for sequence feature prediction that includes homology information. *Bioinformatics* 21(suppl_1):i251-i257.

25. Viklund H, Bernsel A, Skwark M, & Elofsson A (2008) SPOCTOPUS: a combined predictor of signal peptides and membrane protein topology. *Bioinformatics* 24(24):2928-2929.

26. Bernhofer M, Kloppmann E, Reeb J, & Rost B (2016) TMSEG: Novel prediction of transmembrane helices. *Proteins: Struct. Funct. Genet.* 84(11):1706-1716.

27. Molday LL & Molday RS (2014) 1D4: A Versatile Epitope Tag for the Purification and Characterization of Expressed Membrane and Soluble

Proteins. *Protein Affinity Tags: Methods and Protocols*, eds Giannone RJ & Dykstra AB (Springer New York, New York, NY), pp 1-15.

28. Ito C*, et al.* (2018) Deletion of *Eqtn* in mice reduces male fertility and sperm–egg adhesion. *Reproduction* 156(6):579-590.

29. Lin Y-N, Roy A, Yan W, Burns KH, & Matzuk MM (2007) Loss of Zona Pellucida Binding Proteins in the Acrosomal Matrix Disrupts Acrosome Biogenesis and Sperm Morphogenesis. *Mol. Cell. Biol.* 27(19):6794-6805.

30. Li H*, et al.* (2006) TreeFam: a curated database of phylogenetic trees of animal gene families. *Nucleic Acids Res.* 34:D572-D580.

31. Ruan J*, et al.* (2007) TreeFam: 2008 Update. *Nucleic Acids Res.* 36(suppl_1):D735-D740.

32. Gasteiger E*, et al.* (2005) Protein Identification and Analysis Tools on the ExPASy Server. *The Proteomics Protocols Handbook*, ed Walker JM (Humana Press, Totowa, NJ), pp 571-607.

33. Sherman BT*, et al.* (2022) DAVID: a web server for functional enrichment analysis and functional annotation of gene lists (2021 update). *Nucleic Acids Res.* 50(W1):W216–W221.

34. Bräuer P*, et al.* (2019) Structural basis for pH-dependent retrieval of ER proteins from the Golgi by the KDEL receptor. *Science* 363(6431):1103-1107.

35. Omura T (1998) Mitochondria-Targeting Sequence, a Multi-Role Sorting Sequence Recognized at All Steps of Protein Import into Mitochondria. *J. Biochem.* 123(6):1010-1016.

36. Taneja TK, Ma D, Kim BY, & Welling PA (2018) Golgin-97 Targets Ectopically Expressed Inward Rectifying Potassium Channel, Kir2.1, to the trans-Golgi Network in COS-7 Cells. *Front. Physiol.* 9:1070.

37. Ikawa M*, et al.* (2011) Calsperin Is a Testis-specific Chaperone Required for Sperm Fertility. *J. Biol. Chem.* 286(7):5639-5646.

38. Tokuhiro K, Ikawa M, Benham AM, & Okabe M (2012) Protein disulfide isomerase homolog PDILT is required for quality control of sperm membrane protein ADAM3 and male fertility. *Proc. Natl. Acad. Sci. U. S. A.* 109(10):3850-3855.
